# Abundant extrasynaptic expression of α3β4-containing nicotinic acetylcholine receptors in the medial habenula–interpeduncular nucleus pathway in mice

**DOI:** 10.1038/s41598-024-65076-3

**Published:** 2024-06-20

**Authors:** Asuka Tsuzuki, Miwako Yamasaki, Kohtarou Konno, Taisuke Miyazaki, Norio Takei, Susumu Tomita, Michisuke Yuzaki, Masahiko Watanabe

**Affiliations:** 1 Department of Anatomy, Graduate School of Medicine, Hokkaido University, Sapporo, 060-8638 Japan; 2 Department of Anatomy, Faculty of Medicine, Hokkaido University, Sapporo, 060-8638 Japan; 3 Department of Functioning and Disability, Faculty of Health Sciences, Hokkaido University, Sapporo, 060-8638 Japan; 4 Institute for Animal Experimentation, Faculty of Medicine, Hokkaido University, Sapporo, 060-8638 Japan; 5 Department of Cellular and Molecular Physiology, Department of Neuroscience, and Kavli Institute for Neuroscience, Yale University School of Medicine, New Haven, CT 06520 USA; 6 Department of Physiology, School of Medicine, Keio University, Tokyo, 160-8582 Japan

**Keywords:** Neuroscience, Cellular neuroscience, Ion channels in the nervous system, Molecular neuroscience, Synaptic transmission, Anatomy, Nervous system

## Abstract

Nicotinic acetylcholine receptors (nAChRs) in the medial habenula (MHb)–interpeduncular nucleus (IPN) pathway play critical roles in nicotine-related behaviors. This pathway is particularly enriched in nAChR α3 and β4 subunits, both of which are genetically linked to nicotine dependence. However, the cellular and subcellular expression of endogenous α3β4-containing nAChRs remains largely unknown because specific antibodies and appropriate detection methods were unavailable. Here, we successfully uncovered the expression of endogenous nAChRs containing α3 and β4 subunits in the MHb–IPN pathway using novel specific antibodies and a fixative glyoxal that enables simultaneous detection of synaptic and extrasynaptic molecules. Immunofluorescence and immunoelectron microscopy revealed that both subunits were predominantly localized to the extrasynaptic cell surface of somatodendritic and axonal compartments of MHb neurons but not at their synaptic junctions. Immunolabeling for α3 and β4 subunits disappeared in α5β4-knockout brains, which we used as negative controls. The enriched and diffuse extrasynaptic expression along the MHb–IPN pathway suggests that α3β4-containing nAChRs may enhance the excitability of MHb neurons and neurotransmitter release from their presynaptic terminals in the IPN. The revealed distribution pattern provides a molecular and anatomical basis for understanding the functional role of α3β4-containing nAChRs in the crucial pathway of nicotine dependence.

## Introduction

Nicotinic acetylcholine receptors (nAChRs) are widely distributed in the peripheral and central nervous systems and are involved in diverse brain functions. Upon binding the endogenous neurotransmitter acetylcholine or nicotine from smoking, nAChRs allow cations to flow through their channels, thereby modulating neuronal excitability and synaptic transmission^1^. nAChRs in the nervous system consist of eight α subunits (α2–7, α9, α10) and three β subunits (β2–4). The specific stoichiometry of α and β subunits confers distinct pharmacological and biophysical properties to nAChRs, including differences in sensitivity to endogenous and exogenous ligands, as well as in the kinetics and amplitude of ion currents through the channels^2^. In addition, each subunit is expressed in distinct but overlapping patterns in many cell types throughout the brain^3,4^, suggesting specific roles for nAChR subtypes in different brain regions.

While most nAChRs are characterized by rapid desensitization, α3β4-containing receptors show exceptionally slow desensitization^5^ and function as the primary receptors in smokers with chronically high blood nicotine levels^6^. Genome-wide association studies show that single-nucleotide polymorphisms in the *CHRNΑ5-CHRNΑ3-CHRNΒ4* gene cluster, which encodes α5, α3, and β4 nAChR subunits, reduce the aversive properties of nicotine and heighten the risk of excessive smoking^7–9^. Overexpression of the β4 subunit in the medial habenula–interpeduncular nucleus (MHb–IPN) pathway augments α3β4 receptor-mediated currents and increases aversion to nicotine, while a decrease in these currents attenuates nicotine aversion^10^. Conversely, viral-mediated α3 knockdown in the rat MHb or IPN increases nicotine intake^11^. These studies collectively suggest that the normal functioning of α3β4-containing receptors in the MHb–IPN pathway is essential for nicotine avoidance behaviors, which protect against the risk of developing nicotine dependence^12^.

The MHb–IPN pathway plays a critical role in regulating the avoidance of noxious stimuli and adaptive behaviors^13,14^. The MHb is composed of diverse cell types and can be divided into the dorsal and ventral subregions^15,16^. The ventral region (vMHb) comprises approximately two-thirds of the nucleus and consists of neurons that co-release acetylcholine and glutamate^17^, while the dorsal region (dMHb) consists of neurons that co-release glutamate and substance P. Their inputs and outputs are also distinct, with the vMHb receiving glutamatergic input primarily from the triangular septal nucleus (TS), and the dMHb receiving it from the bed nucleus of the anterior commissure^14^. Neurons in the dMHb project to the lateral subnucleus of the IPN (IPL), while those in the vMHb project to the rostral (IPR), central (IPC) and intermediate (IPI) subnucleus of the IPN^18^. Numerous gamma-aminobutyric acid (GABA)ergic neurons within the IPN project to the median and dorsal raphe nuclei^19–21^, where they modulate the activity of serotonergic neurons^22^.

The MHb–IPN pathway regulates the activity of neuromodulatory systems and plays a critical role in anxiety and aversive behaviors^13,23^. In situ hybridization and pharmacological studies^24–27^, as well as studies using transgenic mice^10,28,29^, have revealed abundant cellular expression of α3 and β4 subunits in the MHb–IPN pathway. However, because of the lack of specific antibodies for immunohistochemistry, little is known about the cellular and subcellular expression of endogenous nAChRs in the brain. In the present study, we generated antibodies specific to α3 and β4 nAChR subunits and examined their localization in the MHb–IPN pathway of adult mice.

## Results

### Expression of α3 and β4 mRNAs in the MHb–IPN pathway

**Figure 1:**
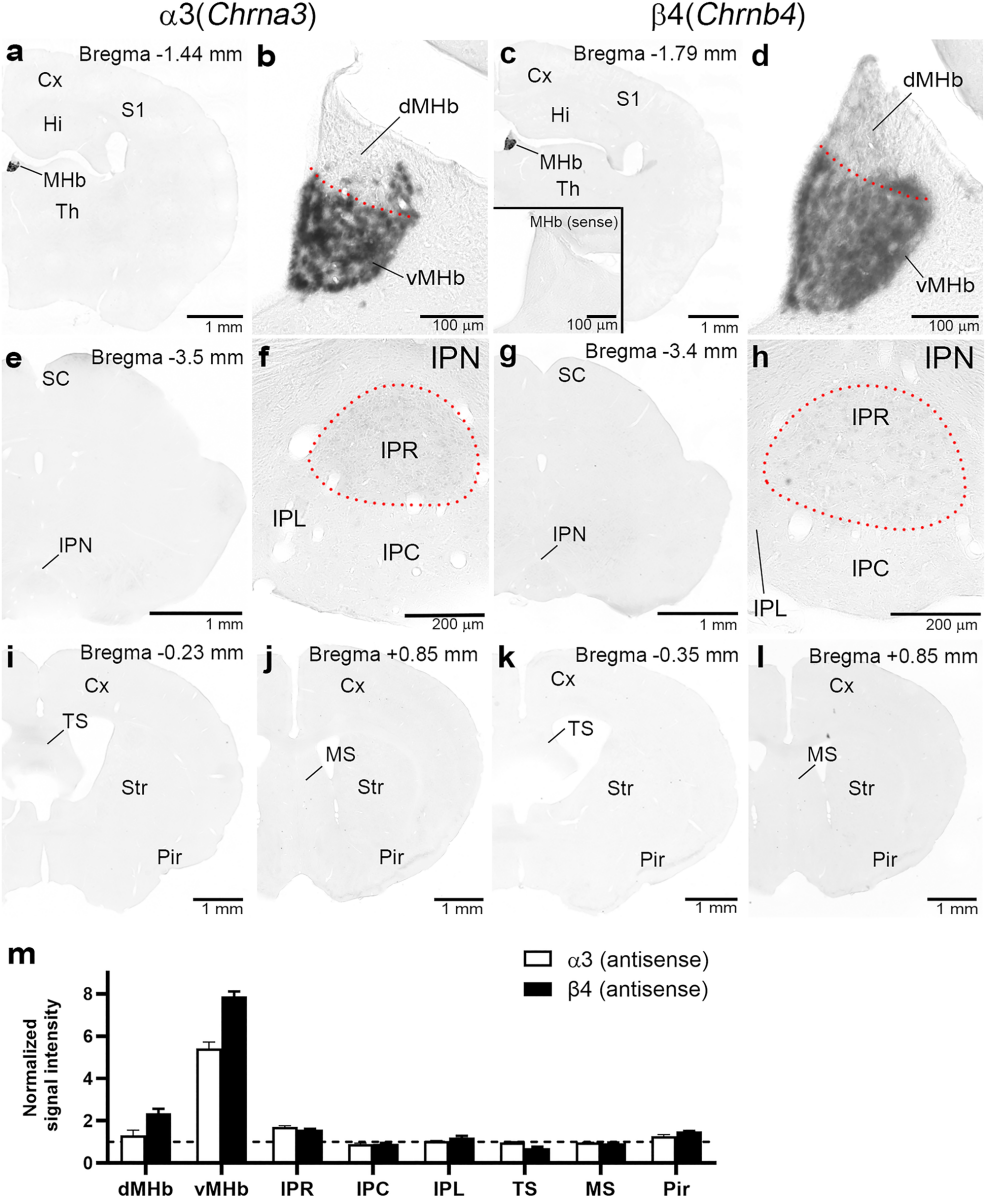
Chromogenic in situ hybridization for α3 and β4 mRNAs. Coronal brain sections hybridized with antisense riboprobes for α3 (**a, b, e, f, i, j**) and β4 (**c, d, g, h, k, l**). An inset in (**c**) shows a negative control using the β4 sense probe. (**a–d**) Sections through the medial habenula (MHb). (**a**) and (**c**) are enlarged in (**b**) and (**d**), respectively. Note that intense signals are mostly confined to the ventral MHb (vMHb), below a red dotted line. (**e**–**h**) Sections through the interpeduncular nucleus (IPN). (**e**) and (**g**) are enlarged in (**f**) and (**h**), respectively. Note that weak signals are confined to the IPR (circled with a red dotted line). (**i, k**) Sections through the triangular septal nucleus (TS). (**j, l**) Sections through the medial septal nucleus (MS). The approximate distance from the Bregma is indicated at the top. (**m**) Bar graphs showing the relative intensities of α3 and β4 mRNA signals in respective nuclei and cortical areas. After converting color images to grayscale, the relative intensities of α3 and β4 mRNA were calculated from the measured gray levels (in arbitrary units, A.U.) and the areas in specified nuclei or regions. These values were then normalized to the intensity in layer II/III of the primary somatosensory cortex (S1), indicated by a dashed line. n = 3 sections (from 3 mice). Data are presented as mean ± SEM. Other abbreviations: Cx, cerebral cortex; dMHb, dorsal MHb; Hi, hippocampus; IPC, central subnucleus of the IPN; IPL, lateral subnucleus of the IPN; IPR, rostral subnucleus of the IPN; Pir, piriform cortex; SC, superior colliculus; Str, striatum; Th, thalamus.

We first examined the expression of mRNAs encoding nAChR α3 and β4 subunits in the MHb–IPN pathway by chromogenic in situ hybridization (Fig. 1). Consistent with previous studies using radiolabeled probes^24,26,27,30^, chromogenic detection using the digoxigenin (DIG)-labeled antisense riboprobe revealed that α3 and β4 mRNAs were prominently expressed throughout the entire vMHb, while their presence in the dMHb was almost restricted to the lateral region (Fig. 1a–d). No significant labeling was detected using the control sense probe for β4 mRNA (Fig. 1c, inset). In the IPN, faint labeling for α3 and β4 mRNAs was observed exclusively in the IPR, while no labeling was detected in the IPC (Fig. 1e–h). In addition, α3 and β4 mRNAs were undetectable in other brain regions, including the TS (Fig. 1i,k) and the medial septal nucleus (MS) (Fig. 1j,l), both of which provide inputs to the vMHb^14,31^. To validate the distinct regional expression patterns, we quantified and normalized the average signals in each region relative to layer II/III of the somatosensory cortex (S1) (Fig. 1m). The measurements showed strong expression of α3 and β4 mRNAs in the vMHb with a normalized intensity above 5. Regions with normalized intensity above 1, such as β4 in the dMHb and α3 and β4 in the IPR, had detectable but weak average signals. Other regions had low signals, similar to S1, where negligible expression has been reported^26,30^. These data confirmed exceptionally high expression in the vMHb and relatively weak expression of α3 and β4 mRNAs in the IPR and dMHb.

**Figure 2:**
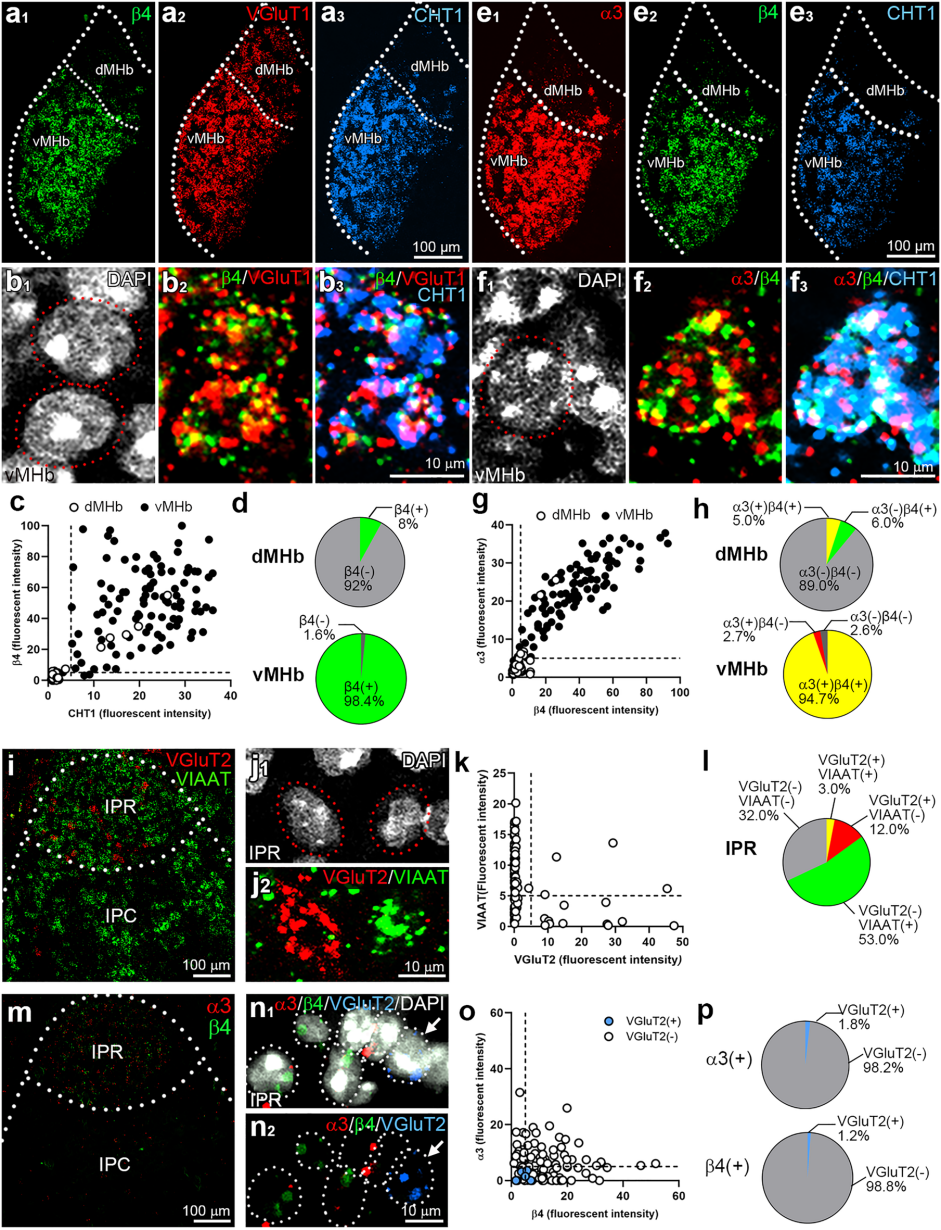
Fluorescent in situ hybridization for α3 and β4 subunit mRNAs with neurochemical markers in the MHb and IPN. (**a**) Triple fluorescent in situ hybridization (FISH) for β4 (green), VGluT1 (red), and CHT1 (blue) in the MHb (Bregma − 1.79 mm). (**b**) Higher magnification images show DAPI-stained individual cells (**b**_1_, circled with a red dotted line) co-express β4, VGluT1, and CHT1 (**b**_2_, **b**_3_). (**c**) A scatterplot of average fluorescent intensities for CHT1 (abscissa) vs. β4 (ordinate) in individual VGluT1-positive cells. Cells from the vMHb are represented by filled dots (n = 100 cells/2 sections/2 mice), and those from the dMHb are represented by open dots (n = 100 cells/2 sections/2 mice). (**d**) The composition of dMHb (top) and vMHb (bottom) cells, calculated from (**c**). (**e**) Triple FISH for α3 (red), β4 (green) and CHT1 (blue) in the MHb. (**f**) A DAPI-stained cell in the vMHb (**f**_1_, circled with a red dotted line) co-expresses α3, β4, and CHT1 (**f**_2_, **f**_3_). (**g**) A scatterplot of average fluorescent intensities for β4 (abscissa) vs. α3 (ordinate) in the MHb. Cells from the vMHb are represented by black circles (n = 150 cells/2 sections/2mice), and those from the dMHb are represented by open circles (n = 100 cells/2 sections/2 mice). (**h**) The composition of dMHb (top) and vMHb (bottom) cells, calculated from (**g**). (**i, j**_**2**_) Double FISH for VGluT2 (red) and VIAAT (green) in the IPN (Bregma -3.5 mm). (**j**_1_) DAPI-stained individual IPR cells (circled with red dotted lines). (**k**) A scatterplot of average fluorescent intensities for VGluT2 (abscissa) vs. VIAAT (ordinate) in the IPR cells (n = 100 cells/2 sections/2 mice). (**l**) The composition of IPR neurons expressing VGluT2 and/or VIAAT. (**m**) Double FISH for α3 (red), and β4 (green) in the IPN. (**n**) Triple FISH for α3 (red), β4 (green), and VGluT2 (blue) and DAPI-staining (white). White dotted lines delineate individual IPR cells. (**o**) A scatterplot of average fluorescent intensities for β4 (abscissa) vs. α3 (ordinate) in the IPR cells. Individual VGluT2-positive cells are represented by blue circles (n = 7 cells/2 sections/2 mice), and -negative cells (n = 103 cells/2 sections /2 mice) are presented by open circles. (**p**) The composition of α3 (top) and β4 (bottom) expressing cells calculated from (**o**).

We then performed highly sensitive multiplex fluorescent in situ hybridization (FISH) using RNAscope to examine cellular expression profiles (Fig. 2). Strong β4 mRNA signals were detected in the vMHb (Fig. 2a_1_), where mRNAs for the glutamatergic neuronal marker, type 1 vesicular glutamate transporter (VGluT1) (Fig. 2a_2_) and the cholinergic neuronal marker, high-affinity choline transporter-1 (CHT1) (Fig. 2a_3_), were also expressed. At higher magnification, β4 mRNA was detected in neurons co-expressing VGluT1 and CHT1 mRNAs (Fig. 2b). Signals for β4 mRNA were detected in 98.4% of vMHb neurons co-expressing CHT1 and VGluT1 mRNAs (Fig. 2c,d, bottom). In contrast, signals for β4 mRNA were detected in a limited number of cells in the dMHb, which expressed VGluT1 mRNA but not CHT1 mRNA (Fig. 2a,c,d, top). Additionally, α3 mRNA was co-expressed with β4 mRNA in the vMHb, detected in 94.7% of neurons expressing CHT1 mRNA (Fig. 2e–g,h, bottom). However, these co-expressing neurons were rarely found in the dMHb (Fig. 2h, top). The IPR contains glutamatergic neurons expressing type 2 vesicular glutamate transporter (VGluT2) and GABAergic neurons expressing vesicular inhibitory amino acid transporter (VIAAT)^28^. Double FISH for VGluT2 and VIAAT (Fig. 2i–l) revealed that VGluT2-negative neurons constituted the major population of IPR cells, accounting for 85.0% (Fig. 2l). Triple FISH revealed that low but discrete signals for α3 and β4 mRNAs were predominantly detected in VGluT2-negative neurons (Fig. 2m–p). These findings indicate robust expression of α3 and β4 subunit mRNAs in glutamatergic/cholinergic neurons in the vMHb, and weak expression in glutamatergic neurons in the dMHb and VGluT2-negative (most likely, GABAergic) neurons in the IPR.

### Distribution of α3 and β4 subunit proteins in the MHb–IPN pathway

**Figure 3:**
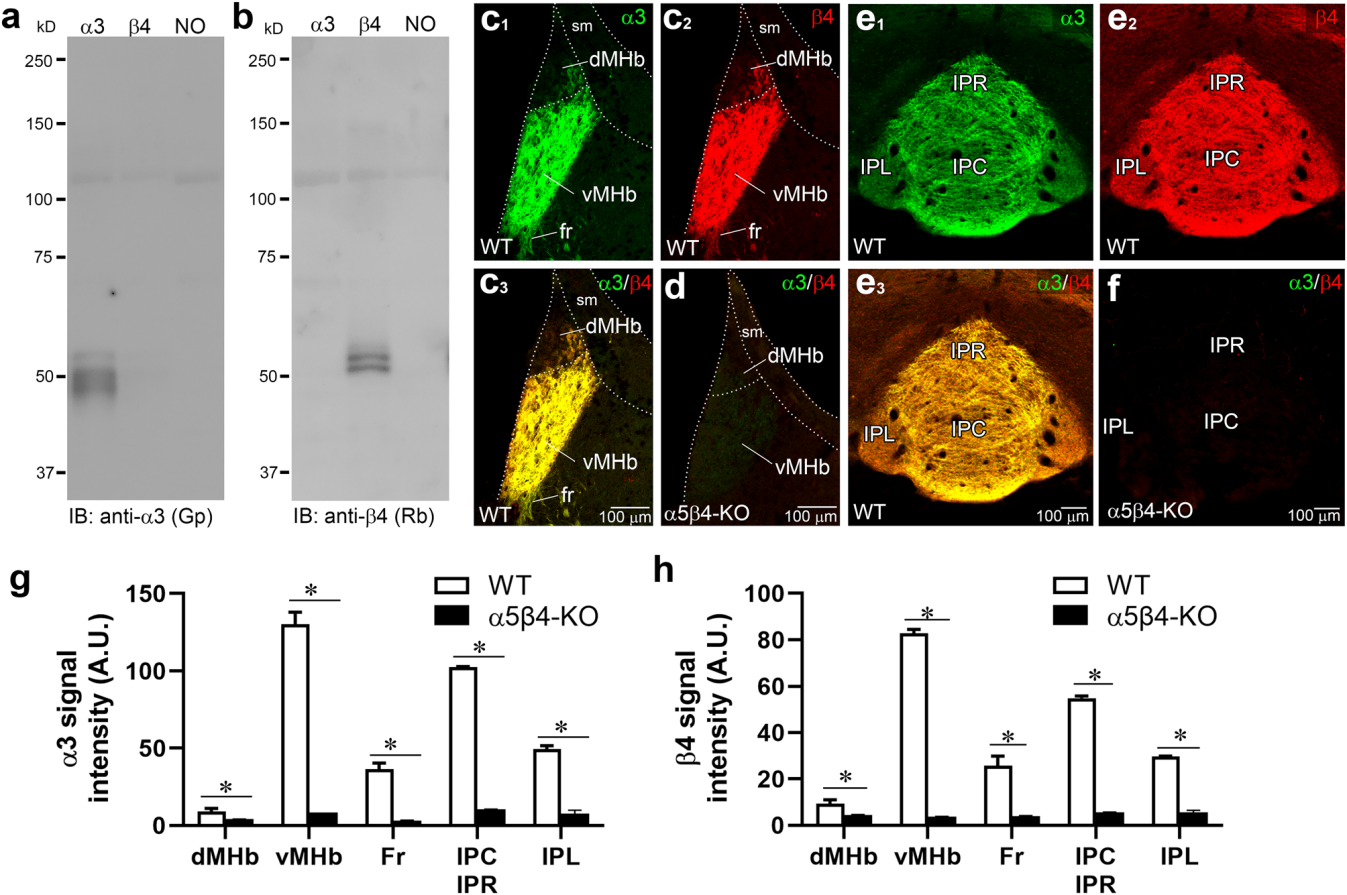
Specificity of α3 and β4 antibodies. (**a, b**) Characterization of α3 and β4 antibodies by immunoblot (IB) experiments. Protein samples from *Xenopus* oocytes injected with corresponding cRNA (1 ng) were separated by 7.5% SDS-PAGE. Original blots are presented in Supplementary Fig. S1. (**c**, **d**) Double immunofluorescence for α3 (green) and β4 (red) in coronal sections through the MHb (Bregma -1.43 mm) in WT (**c**) and α5β4-KO (**d**). In the WT MHb, note the intense labeling in the vMHb and moderate labeling in the fasciculus retroflexus (fr), which projects to the IPN. (**e, f**) Double immunofluorescence for α3 (green) and β4 (red) in the WT (**e**) and α5β4-KO IPN (Bregma -3.5 mm) (**f**). In the WT IPN, intense labeling is observed in the rostral and central subnuclei (IPR and IPC), with relatively weak labeling in the lateral subnucleus (IPL). (**g, h**) Bar graphs comparing the signal intensities, measured in arbitrary gray units (A.U.), of α3 (**g**) and β4 (**h**) immunofluorescent signals in respective nuclei and cortical areas from WT and α5β4-KO mice. n = 6 sections/3 WT and 3 KO mice. The signal intensities in WT and α5β4-KO mice, obtained under identical image acquisition settings, are presented as mean ± SEM. *p < 0.05, Mann–Whitney U-test. sm, stria medullaris.

Next, we examined the expression of α3 and β4 subunits at the protein level by immunohistochemistry. Antibodies were raised against the mouse α3 subunit in the guinea pig and against the mouse β4 subunit in the rabbit. To test their specificity, we expressed mouse α3 and β4 subunits in *Xenopus laevis* oocytes and prepared lysate for immunoblot. Each antibody recognized the corresponding subunit as multiple bands at around 50–57 kDa in lysates from oocytes expressing the corresponding subunit, but not in lysates from oocytes expressing the other subunit or from uninjected oocytes (Fig. 3a,b).

Double immunofluorescence using glyoxal-fixed brain slices from wild-type (WT) mice revealed intense and overlapping signals of α3 and β4 subunits in the vMHb, resulting in a merged yellowish color (Fig. 3c). Additionally, the axon bundles of the habenulo-peduncular tract (fasciculus retroflexus, fr) (Fig. 3c), which originate from the vMHb, and their major projection targets, such as the IPC and IPR (Fig. 3e), showed moderate signals. The IPL (Fig. 3e), which receives projections from dMHb, also showed intermediate labeling. To validate the specificity of these signals, we used α5β4-knockout (KO) mice as a negative control. Since α3-KO mice are lethal^32^ and β4-KO mice were unavailable, we opted for α5β4-double KO mice^33^ as an alternative. Notably, it has been shown that α3 mRNA expression decreases by approximately 50% and 70% in β4-KO and α5β4-KO mice, respectively^33^. Based on these findings, we considered α5β4-KO mice to be appropriate as a negative control for not only the β4 subunit antibody but also the α3 subunit antibody. Under the same conditions, signals for both α3 and β4 subunits observed in the MHb–IPN pathway in WT brains almost disappeared in α5β4-KO brains (Fig. 3d,f). Quantitative comparisons of immunostaining between α5β4-KO and WT signals confirmed their significant reductions in the KO group (Fig. 3g,h), thus validating the specificity of immunostainings for α3 and β4 subunits.

### Subcellular localization of α3 and β4 subunits in the vMHb

**Figure 4:**
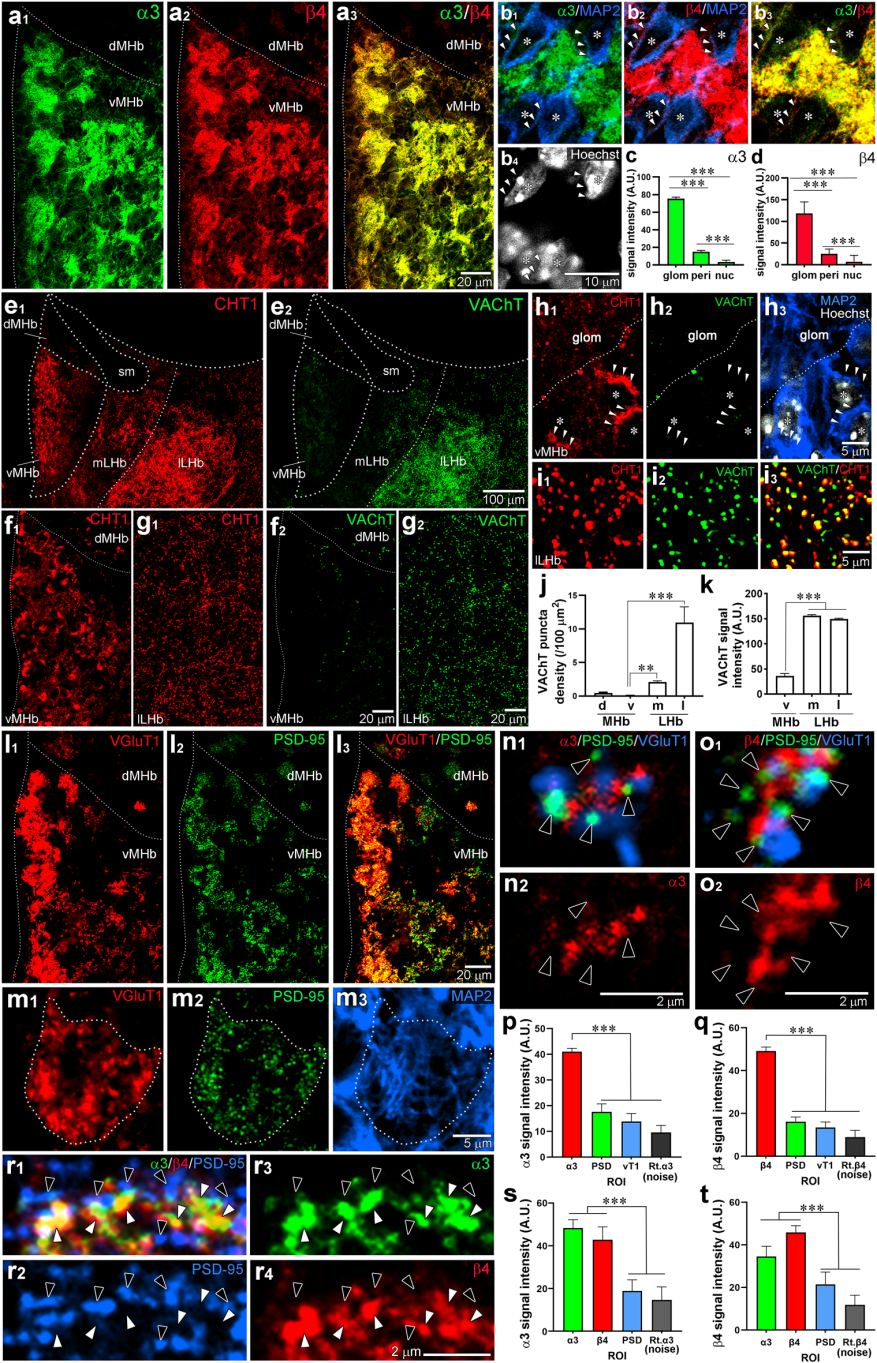
Non-synaptic colocalization of α3 and β4 in the MHb glomerulus. (**a**) Double immunofluorescence for α3 (green) and β4 (red) in the MHb. (**b**) Triple immunofluorescence for α3 (green), β4 (red), and MAP2 (blue) with Hoechst nuclear staining (**b**_4_, white, asterisk). Arrowheads indicate the perikaryon. (**c**, **d**) Bar graphs comparing the immunofluorescent signals of α3 (**c**) and β4 (**d**) in the glomerulus (glom), perikaryon (peri), and nucleus (nuc). (**e, f**) Separate channel images of double immunofluorescence for CHT1 (**e**_1_, **f**_1_, **g**_1_, red) and VAChT (**e**_2_, **f**_2_, **g**_2_, green) in the MHb, medial LHb (mLHb) and lateral LHb (lLHb). (**h**) Separate channel images of triple immunofluorescence for CHT1 (red), VAChT (green), and MAP2 (blue) with Hoechst (white) in the vMHb. The perikaryon and nucleus are indicated by arrowheads and an asterisk, respectively. (**i**) Double immunofluorescence for CHT1 (red) and VAChT (green) in the lLHb. (**j, k**) Bar graphs comparing the densities (**j**) and intensities (**k**) of VAChT-positive puncta in the MHb and LHb. (**l**) Double immunofluorescence for VGluT1 (red) and PSD-95 (green). (**m**) Separate channel images of triple immunofluorescence for VGluT1 (red), PSD-95 (green), and MAP2 (blue) showing the glomerulus (circled with a dotted line) in the vMHb. (**n**, **o**) Triple immunofluorescence for α3 (**n**, red) or β4 (**o**, red) with PSD-95 (green), and VGluT1 (blue) in the vMHb. Black arrowheads indicate PSD-95 labeling. (**p**, **q**) Bar graphs comparing the α3 or β4 signal intensities in the region of interest (ROI), determined by α3 or β4, PSD95, and VGluT1 labeling. (**r**) Triple immunofluorescence for α3 (green), β4 (red), and PSD-95 (blue) in the vMHb. Black arrowheads indicate PSD-95 labeling, and white arrowheads indicate α3 and β4 co-localization. (**s**, **t**) Bar graphs comparing the α3 or β4 signal intensities in the ROI, determined by α3, β4, and PSD-95 labeling. The noise level in (**p**), (**q**), (**s**), and (**t**) was determined using a designated ROI and images from channels rotated 90° (Rt.). ***p < 0.001, **p < 0.01, Kruskal–Wallis test and Dunn’s multiple comparison test. Additional information on the number of samples analyzed and statistical data is provided in Supplemental Table S1.

We next examined the subcellular localization of α3 and β4 subunits in the vMHb by immunofluorescence, employing specific markers (Fig. 4). Intense immunostaining of α3 and β4 subunits was observed in the neuropil region (Fig. 4a), which is also called the glomerulus situated amidst densely packed small cells^34^. By combining microtubule-associated protein 2 (MAP2) immunostaining, a marker for neuronal perikarya and dendrites, with Hoechst nuclear staining, we further observed weak staining for α3 and β4 subunits along the surface of MAP2-positive perikarya (Fig. 4b, arrowheads). Quantitative comparisons confirmed that strong immunofluorescent signals were detected in the glomerulus, and weak signals in the perikaryon, both significantly higher than the signals in the nucleus (Fig. 4c,d). We next examined the vMHb with two cholinergic markers: CHT1, which labels both somatodendritic and axonal domains^35,36^, and vesicular acetylcholine transporter (VAChT), which preferentially labels axon terminals (Fig. 4e–k). While CHT1 labeling was intense in both the vMHb and the entire lateral habenula (LHb) (Fig. 4e1,f1,g1), VAChT labeling was concentrated in the lateral half of the LHb (lLHb) with notably weaker and less frequent labeling in the medial half of the LHb (mLHb) and vMHb. (Fig. 4e2,f2,g2). At higher magnification, CHT1 labeling in the vMHb was prominent in the perikaryon (Fig. 4h1, arrowheads), while only diffuse and faint signals were detected in the glomerulus (Fig. 4h1, the region above the dotted line). Similarly, punctate labeling of VAChT was rare in the vMHb, resulting in a largely blank labeling pattern (Fig. 4h2). In contrast, CHT1 and VAChT labeling appeared as intense puncta, and were nearly completely overlapped in the lLHb (Fig. 4i). Quantitative comparisons confirmed the scarcity of VAChT-positive terminals (Fig. 4j) and low VAChT expression (Fig. 4k) in the vMHb. These results suggest that the LHb receives intense cholinergic innervation, with only sparse innervation in the vMHb.

To examine the glomerulus in more detail, we utilized VGluT1 and postsynaptic density protein 95 (PSD-95) as markers for the glutamatergic presynapse and postsynapse, respectively (Fig. 4l,m). At higher magnification, we found that the glomerulus was crowded with PSD-95 puncta, VGluT1-positive terminals, and MAP2-labeled thin dendrites (Fig. 4m). In triple immunofluorescence for α3 or β4 with PSD-95 and VGluT1, neither subunit within the glomerulus overlaps with VGluT1 or PSD-95 puncta; instead, both subunits were clustered between, or close to, them (Fig. 4n,o). To confirm this observation, we measured the signal intensities of α3 or β4 in the glomerulus and examined their overlap with PSD-95 and VGluT1 using triple immunofluorescence (Fig. 4p,q). To this end, we defined the region of interest (ROI) based on α3- or β4-, PSD-95-, and VGluT1-positive puncta. In addition, we measured the noise levels by applying the ROI for the α3 or β4 signals to the images where the α3 or β4 channels were rotated by 90°, respectively. We observed that α3 and β4 signals overlapping with PSD-95 and VGluT1 signals were comparable to the noise levels, whereas the remaining α3 and β4 signals exhibited significantly higher levels than the noise levels (Fig. 4p,q). Triple immunofluorescence for α3, β4, and PSD-95 showed good overlap between α3 and β4 (Fig. 4r, white arrowheads), but not with PSD-95 (Fig. 4r, black arrowheads). To confirm this observation, we measured the signal intensities of α3 and β4 and examined their overlap with PSD-95 using triple immunofluorescence (Fig. 4s, t). The quantitative comparison of the signal intensities revealed that the α3 and β4 signals were similar and well-matched, but those overlapping with PSD-95 were comparable to the noise levels (Fig. 4s,t). These results suggest that, at synaptic glomeruli in the vMHb, α3 and β4 subunits are predominantly co-expressed in the extrasynaptic regions.

**Figure 5:**
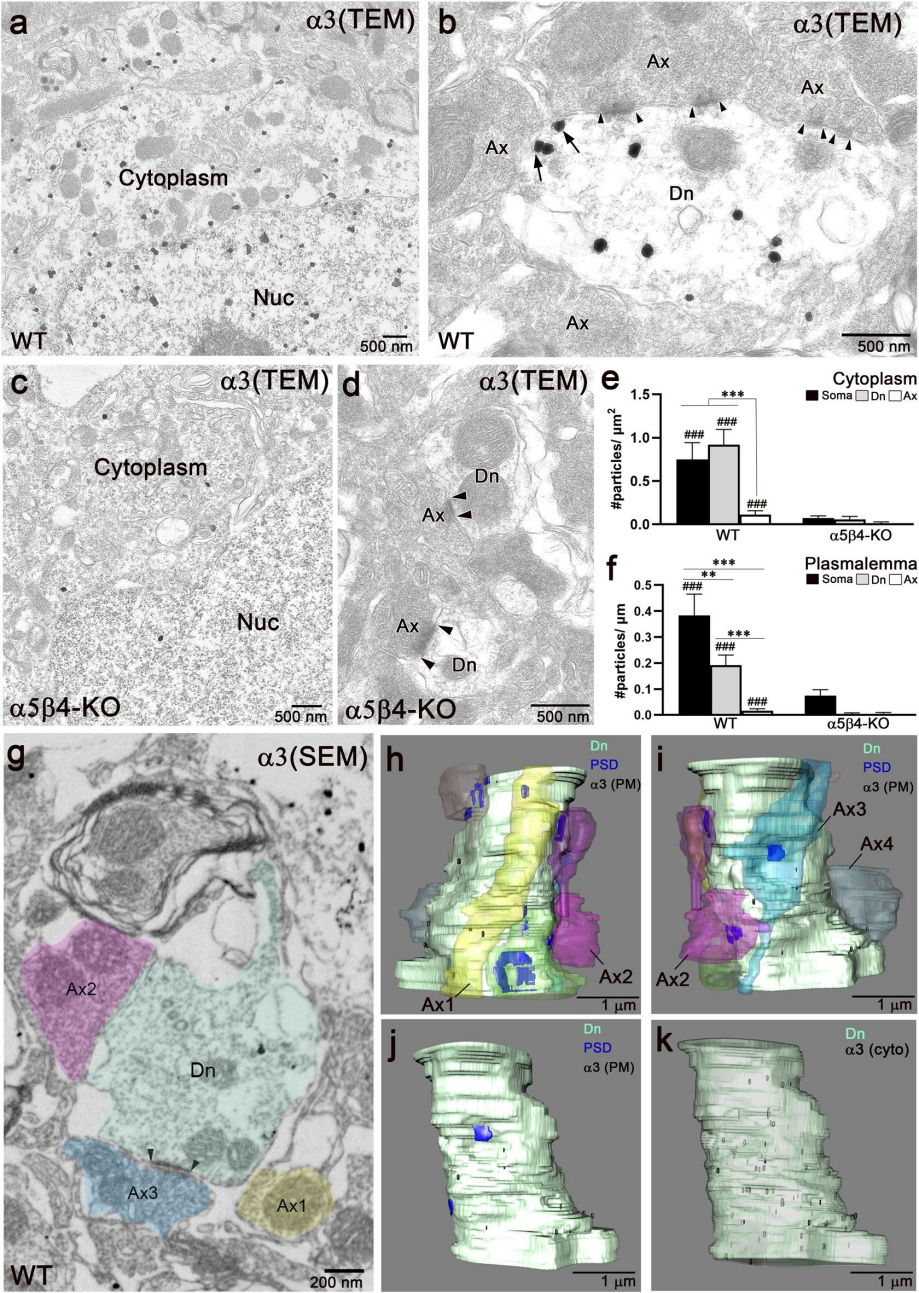
Pre-embedding immunoelectron microscopy for α3 in the vMHb. (**a–d**) Pre-embedding immunoelectron microscopy for the α3 subunit acquired with a transmission electron microscope (TEM) in the vMHb from WT (**a**, **b**) and α5β4-KO (**c**, **d**) mice. In WT, metal particles for the α3 subunit are observed in the soma (**a**) and dendrites (Dn, **b**), but rarely in axons (Ax, **b**). Note that α3 labeling (arrows) is not associated with the postsynaptic density, which is flanked by arrowheads. Labeling in the cytoplasm and plasmalemma is almost absent in the α5β4-KO mice (**c**, **d**). (**e**) Summary bar graphs showing the cytoplasmic labeling in subcellular compartments in WT and α5β4-KO mice. The number of profiles examined, which were used for statistical testing, are: WT (soma, 9; dendrite, 40; axon, 62; from 2 mice); KO (soma, 9; dendrite, 44; axon, 56; from 2 mice). In WT mice, the labeling densities in all compartments are higher than those in α5β4-KO mice (###*p* < 0.001, Mann–Whitney U-test). (**f**) Summary bar graphs comparing the plasmalemma labeling in subcellular compartments in WT and α5β4-KO mice. In WT mice, the labeling densities in all compartments are higher than those in α5β4-KO mice (###*p* < 0.001, Mann–Whitney U-test). The number of analyzed profiles is the same as in (**e**). (**g**) Pre-embedding immunoelectron microscopy for the α3 subunit acquired with a scanning EM (SEM). (**h**–**k**) Partial reconstruction of a vMHb dendrite using consecutive 40 ultrathin section images, including ***g***. A thin dendrite (Dn, green) is contacted by four axons (Ax1–4). Front view (**h**) and back view (**i–k**) of axons facing the postsynaptic density (PSD, blue) show that metal particles representing α3 (black) are sparse on the dendritic plasmalemma (PM) and avoid the PSD (**h–j**), but are abundant in the cytoplasm (cyto, **k**). ***p < 0.001, **p < 0.01, Kruskal–Wallis test and Dunn’s multiple comparison test.

Because the glyoxal fixative has not yet been optimized for pre-embedding immunoelectron microscopy^37^ and does not preserve the integrity of the neuropil, including contacts between dendrites and axons, we employed a low concentration (2%) of paraformaldehyde (PFA) for this purpose. This fixation, however, failed to detect specific signals for the β4 subunit. Thus, we applied the α3 antibody to 2% PFA fixed sections (Fig. 5). In WT mice, metal particles for the α3 subunit were observed in the somatic (Fig. 5a) and dendritic (Fig. 5b) cytoplasm. Moreover, cytoplasmic or plasmalemma labeling was almost absent in α5β4-KO mice (Fig. 5c,d). In WT mice, a fraction of these particles was associated with the dendritic plasmalemma (Fig. 5b, arrows). However, the postsynaptic membrane at asymmetrical synapses remained unlabeled (Fig. 5b, flanked by arrowheads). In all compartments, the densities of metal particles in the cytoplasm and plasmalemma were significantly higher in WT mice compared to α5β4-KO mice (Fig. 5e,f), confirming the specificity of the detected signals. Additionally, the cytoplasmic labeling densities in somas and dendrites were significantly higher than those in axons (Fig. 5e). Similarly, the plasmalemma labeling densities in somas and dendrites were also significantly higher than those in axons (Fig. 5f). In the same compartment, most metal particles for the α3 subunit were cytoplasmic [84.5 ± 4.6% in somas (n = 9 profiles; 855 particles, 2 mice); 60.7 ± 7.1% in dendrites (n = 40 profiles; 360 particles, 2 mice); 50.0 ± 16% in axons/terminals (n = 62 profiles; 228 particles, 2 mice)]. This ratio did not significantly differ among the compartments (*p* = 0.66, Kruskal–Wallis test).

We further examined the subcellular distribution of the α3 subunit by three-dimensional (3D) reconstruction of serial immunoelectron microscopic images acquired with a scanning electron microscope (SEM) (Fig. 5g–k). A partial reconstruction of a dendritic segment (3.6 µm) revealed that four to five axons were associated with a thin dendrite, forming asymmetrical synapses (Fig. 5g–i). Metal particles corresponding to the α3 subunit were scattered across the extrasynaptic plasmalemma, without showing a tendency to cluster at specific sites (Fig. 5h–j). Additionally, more metal particles were observed in the cytoplasm (Fig. 5k) than on the plasmalemma (Fig. 5h–j). These results suggest that the α3 subunit is widely distributed in the cytoplasm and on the extrasynaptic cell surface of the soma and dendrites of vMHb neurons.

### Subcellular localization of α3 and β4 subunits in the IPN

**Figure 6:**
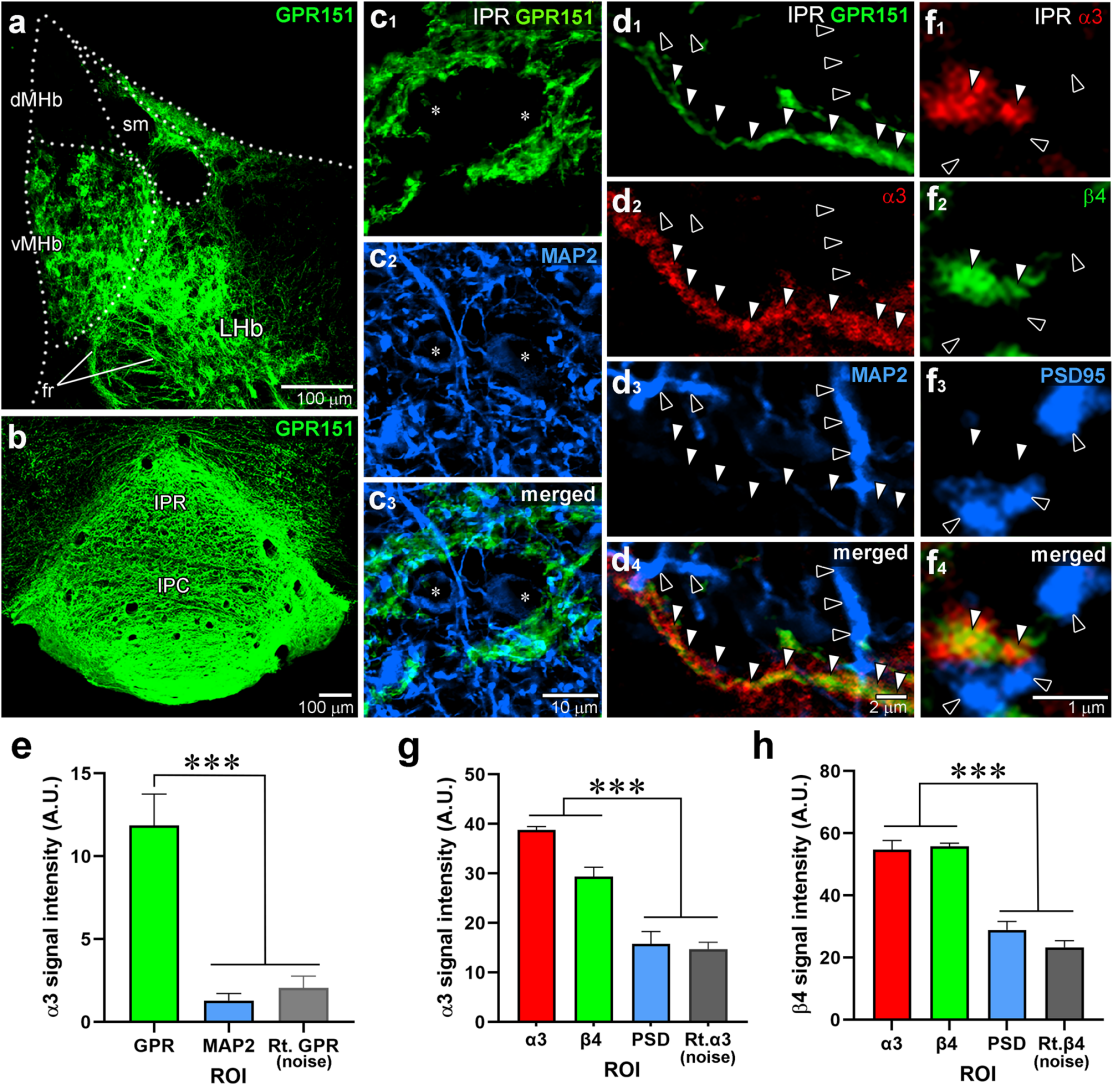
Non-synaptic colocalization of α3 and β4 in cholinergic axons in the IPN. (**a, b**) Immunofluorescence for GPR151 (green) in the MHb (**a**) and the IPN (**b**). (**c**) Double immunofluorescence for GPR151 (green) and MAP2 (blue) distinguishes axons from the MHb and the soma (asterisk) and dendrites of IPR neurons. (**d**) Triple immunofluorescence for α3 (red), GPR151 (green), and MAP2 (blue) shows that α3 labeling is observed along GPR151-positive MHb axons (white arrowheads), but not in MAP2-positive IPR dendrites (black arrowheads). (**e**) Bar graphs comparing the α3 signal intensities in the ROI, determined by GPR151 and MAP2 labeling in triple immunofluorescence images. (**f**) Triple immunofluorescence for β4 (green), α3 (red), and PSD-95 (blue). The α3 and β4 labeling show good overlap (white arrowheads), but neither overlaps with the PSD-95 labeling (black arrowheads). (**g, h**) Bar graphs comparing the α3 (**g**) and β4 (**h**) signal intensities in the ROI, determined by α3, β4, and PSD-95 labeling from triple immunofluorescence images. The noise level was determined by applying the indicated ROI to the 90º rotated (Rt.) α3 (**e**, **g**) or β4 (**h**) channel images. In (***e***), the α3 signal intensity in the MAP2-defined ROI is comparable to the noise level (*p* = 0.99). In (***g***) and (***h***), the α3 and β4 signal intensity in the PSD-95-defined ROI is comparable to the noise level (*p* = 0.99 and *p* = 0.10, respectively). Statistical significance was assessed using Kruskal–Wallis test and Dunn’s multiple comparison test. ***p < 0.001, **p < 0.01. Additional information on the number of samples analyzed and statistical data is provided in Supplemental Table S2.

Finally, we examined the subcellular localization of α3 and β4 subunits in the IPN. Immunofluorescence for GPR151, an orphan G protein-coupled receptor used as a marker of habenular axons^38,39^, revealed MHb axons projecting to the IPN (Fig. 6a,b). Double immunofluorescence for MAP2 and GPR151 showed that the MAP2-positive dendrites intersect with GPR151-positive habenular axons in the IPR (Fig. 6c). Triple immunofluorescence for MAP2, GPR151 and α3 showed that the α3 subunit was localized in GPR151-positive habenular axons (Fig. 6d, white arrowheads), but not in MAP2-positive dendrites of IPR neurons (Fig. 6d, black arrowheads). To examine the expression of α3 in GPR151- or MAP2-labeled structures, we defined the ROI based on GPR151 and MAP2 labeling and measured the α3 signal intensities and the noise levels using triple immunofluorescence (Fig. 6d,e). We found that α3 signals overlapping with MAP2 were comparable to the noise levels, while those overlapping with GPR151 were significantly higher than the noise levels (Fig. 6e). Triple immunofluorescence for α3, β4, and PSD-95 showed good overlap between α3 and β4 subunits (Fig. 6f, white arrowheads) but not with PSD-95 puncta (Fig. 6f, black arrowheads). Measurements of signal intensities revealed that the α3 and β4 signals were similar and well-matched, while those overlapping with PSD-95 were comparable to the noise levels (Fig. 6g,h). These results suggest that α3 and β4 subunits are expressed at habenular axons, but not in the postsynaptic elements in the IPR.

**Figure 7:**
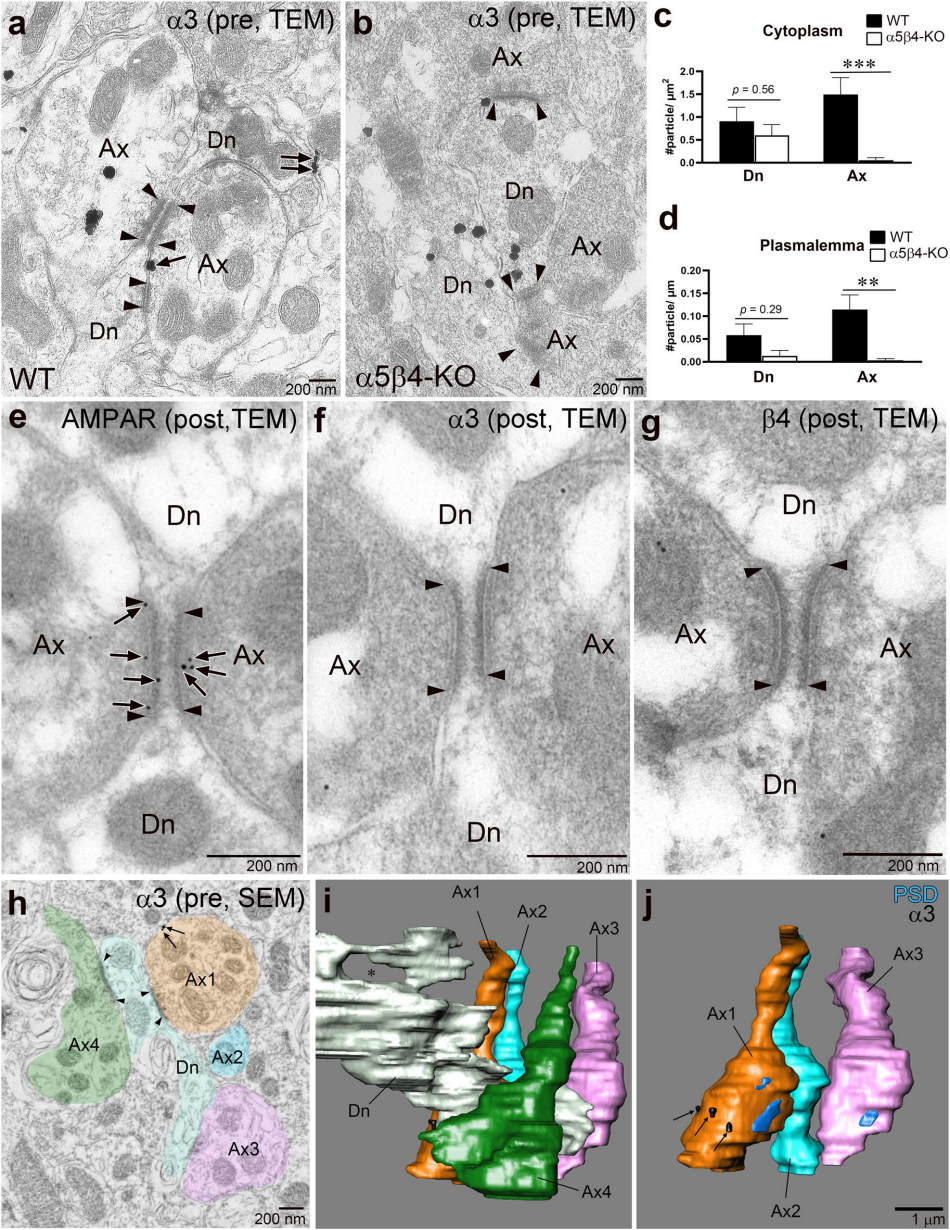
Pre- and post-embedding immunoelectron microscopy for α3 in the IPN. (**a, b**) Pre-embedding immunoelectron microscopy for the α3 subunit acquired with TEM. In WT mice, α3 labeling is observed on the plasmalemma (arrows) and in the cytoplasm of axon terminals (Ax, **a**). Note that plasmalemma α3 labeling (arrows) is not associated with the postsynaptic density (flanked by arrowheads). In α5β4-KO mice, cytoplasmic and plasmalemma labeling in dendrites is almost completely absent, but the labeling in the dendrites (Dn, b) persists. (**c, d**) Summary bar graphs comparing cytoplasmic labeling (**c**) and plasmalemma labeling (**d**) in subcellular compartments in WT and α5β4-KO mice. The cytoplasmic and plasmalemma labeling in dendrites of WT and α5β4-KO mice are comparable (*p* = 0.56 and *p* = 0.29, respectively; Mann–Whitney U-test). The cytoplasmic and plasmalemma labeling in axons are significantly decreased in α5β4-KO mice compared to WT mice (***p < 0.001, **p < 0.01; Mann–Whitney U-test). In (**c**) and (**d**), the number of profiles examined, which were used for statistical testing, are: WT (dendrite, 27; axon, 32; from 2 mice); KO (dendrite, 18; axon, 29; from 2 mice). (**e–g**) Post-embedding immunogold EM for AMPAR (**e**), α3 (**f**), and β4 (**g**). Note that while immunogold labeling for AMPARs (**e**, arrows) is abundant on the PSD (flanked by arrowheads), labeling for α3 and β4 is not detected on the PSD (flanked by arrowheads; **f, g**). (**h–j**) Pre-embedding immunoelectron microscopy for α3 acquired with a SEM (**h**) and partial reconstruction of axons and dendrites in the IPN using consecutive 50 ultrathin section images, including (**h**)*.* (**i**) A dendritic protrusion contacted by axons (Ax1–4) forms a characteristic hollow (asterisk). (**j**) An alternate perspective of the reconstruction, removing Ax4 and a dendritic protrusion from (**i**), shows that the dendritic protrusion forms multiple synapses (PSD, blue) with axons (Ax1 and Ax3), and α3 labeling (arrows) is sparse on the axonal surface.

Pre-embedding immunoelectron microscopy using 2% PFA-fixed brain sections detected the α3 subunit in dendrites and axons/terminals in WT mice (Fig. 7a). We frequently observed "crest-type" synapses characterized by prominent postsynaptic thickening and parallel alignments of two apposing synapses (Fig. 7a). In dendrites, the α3 subunit was detected in the cytoplasm and on the plasmalemma but not on the postsynaptic or presynaptic membranes of crest-type synapses (Fig. 7a). The labeling densities in the dendritic cytoplasm and plasmalemma in WT and α5β4-KO mice were similar, suggesting low levels or nonspecific labeling in dendrites by immunoelectron microscopy. In contrast, significantly higher labeling density was observed in the axonal plasmalemma, and cytoplasm of WT mice compared with α5β4-KO mice (Fig. 7c,d). In the IPN, the α3 subunit was mainly localized to the cytoplasm of axons (72.5 ± 9.3%; n = 32 profiles; 76 particles, 2 mice), with the remainder expressed at the extrasynaptic axonal surface. Furthermore, we tested the synaptic expression of α3 and β4 subunits at crest-type synapses by post-embedding immunoelectron microscopy. While AMPA-type glutamate receptors (AMPAR) were readily detected at the synapse (Fig. 7e), α3 or β4 subunits were not observed (Fig. 7f,g). These electron microscopic findings suggest that α3 and β4 subunits are preferentially targeted to axons and extrasynaptic terminals at IPN synapses. Because synapses between MHb axons and IPN neurons are morphologically complex, we made 3D reconstructions of serial immunoelectron microscopic images acquired with a SEM. A partial reconstruction of IPN dendrites showed that they had numerous protrusions that formed unique crest-type synapses with adjacent axons (Fig. 7h,i, Ax1 and Ax4). We observed that more than five axons running along a thin dendrite formed *en passant* asymmetrical synapses (Fig. 7i,j). Like in the MHb, metal particles for the α3 subunit were scattered on the extrasynaptic plasmalemma without clustering at any particular site. This reconstruction confirmed the extrasynaptic distribution of the α3 subunit in axons traversing the IPN.

## Discussion

MHb neurons have the highest nAChR expression in the brain. In situ hybridization and pharmacological studies have shown that α3β4-containing receptors are exclusively expressed in this pathway. Their key roles in nicotine aversion and smoking behaviors have been revealed by genome-wide association studies and studies using gene manipulation strategies. In the present study, we demonstrated abundant α3 and β4 subunit expression in the MHb–IPN pathway. Moreover, we showed, for the first time, unique cellular and subcellular distribution patterns of endogenous α3 and β4 subunits in this pathway.

Immunohistochemical detection of endogenous nAChRs poses significant challenges. Antibodies targeting α3, α4, α7, β2, and β4 subunits often yield nonspecific labeling, as evidenced by persistent immunosignals in corresponding KO mice brains^40,41^. Gahring et al. analyzed expression using another β4 subunit antibody, yet specificity was not confirmed with KO mice^42^. Localization information for nAChRs has been obtained from ligand-binding autoradiography^4^ and reporter expression in green fluorescent protein knock-in and transgenic mice^10,28,29^. We often face difficulties in developing or finding specific antibodies to neural proteins, especially receptors and ion channels that are applicable to immunostaining using fixed brain sections^43^. This seems to arise from the complex and highly ordered structure of the receptor/ion channel molecule itself, and from mandatory interaction with many other pore-forming and auxiliary subunits, and scaffolding proteins for proper function and regulation. From ten antigen peptides immunized in rabbits and guinea pigs, only two specific antibodies were obtained: a guinea pig antibody to the mouse α3 subunit (345–394 amino acid residues) and a rabbit antibody to the mouse β4 subunit (206–230 amino acid residues). We tested the subunit selectivity of the α3 and β4 antibodies by immunoblot using a heterologous expression system (Fig. 3a,b) and immunofluorescence for α3 and β4 subunits in α5β4-KO mice (Fig. 3d,f,g,h). The detection of multiple bands in the present immunoblots (Fig. 3a,b) raises the possibility that these antibodies might recognize not only α3 and β4 subunits but also other molecular entities. Moreover, they could also detect α3 and β4 subunits that have undergone proteolysis and/or differential post-translational modifications. Nevertheless, the specificity of immunohistochemical signals, at least, for β4 subunits was shown by blank labeling in α5β4-KO mice. We utilized α5β4-KO mice as a negative control due to the unavailability of single KO mice for each subunit in the present study. This limitation also stems from the lethality of α3-KO mice due to multi-organ dysfunction^32^, and from the co-location of α5 and β4 genes on the same chromosome. The blank α3 labeling in α5β4-KO mice (Fig. 3d,f,g,h) likely reflects the possible downregulation occurring at both the transcriptional and translational levels. Common transcriptional regulatory mechanisms have been suggested to control the expression of three genes encoding α5, α3, and β4 subunits^44^. Notably, α3 mRNA expression has been demonstrated to decrease by approximately 50% and 70% in β4-KO and α5β4-KO mice, respectively^33^. Considering that α3 and β4 subunits are incorporated into the same channel in the heterologous expression system^45^, it is also possible that the β4 subunit is required for the assembly of α3-containing receptors. Together with the previous pharmacological observations that almost all α3-containing nAChRs in the MHb are α3β4-containing receptors^11,24^, our findings suggest that the α3 signals mainly represent α3β4-containing receptors and that the β4 subunit is required for the formation of α3-containing nAChRs in this pathway.

Multiplex FISH, immunofluorescence and immunoelectron analyses collectively revealed that α3β4-containing nAChRs are highly enriched in the somatodendritic compartment of glutamatergic/cholinergic neurons in the MHb, with negligible labeling in presynaptic inputs. This selective expression is in line with the high transcript levels in the MHb and the lack of transcript signals in the TS and MS (Fig. 1i–l), which provide the major glutamatergic^14^ and cholinergic inputs^31,46^, respectively, to the MHb. Electrophysiological observations support this finding. Application of nicotine to acute MHb slices elicits slow excitatory currents and increases firing frequency, effects that are dampened by α3β4-containing receptor-selective antagonists^24,29^. Furthermore, the focal application of agonists to the soma and dendrites of MHb neurons induces slow excitatory currents^28,47^. Therefore, the primary function of α3β4-containing nAChRs is likely the cholinergic enhancement of the excitability of MHb neurons. Unlike other brain regions that undergo cholinergic enhancement of the excitation, vMHb displays notably weak VAChT immunoreactivity and sparse distribution of VAChT-positive terminals (Fig. 4e–k). This aligns with a previous report describing the low density of VAChT-positive fibers in the MHb^48^. This finding is rather surprising because MHb has been considered to receive major cholinergic innervation from the MS and diagonal band of Broca^49,50^. This discrepancy raises the possibility that nAChRs in the vMHb sense acetylcholine diffusing from the adjacent LHb. The exceptionally high expression levels of these receptors in the somatodendritic compartment may facilitate their potent modulatory function by acetylcholine.

In the present study, we observed dense distribution and moderate immunoreactivity for α3 and β4 subunits in cholinergic axons and terminals originating from the MHb within the IPN. Conversely, in the IPR, where VGluT2-negative neurons predominate, signals for α3 and β4 mRNAs were low (Fig. 2m), and MAP2-positive somatodendritic elements exhibited similarly low or negligible labeling of α3 and β4 subunits (Fig. 6d,e). Immunoelectron microscopy revealed substantial immunolabeling within the axonal compartment. However, dendritic labeling was insignificant when compared to α5β4-KO mice, which were utilized as a negative control (Fig. 7c,d). This observation suggests that α3β4-containing nAChRs are primarily expressed in cholinergic axons and terminals projecting from MHb neurons, with much lower levels observed in somatodendritic elements of IPN neurons.

Excitatory responses by nAChRs are difficult to elicit in IPN neurons^17,51^, and require elevated acetylcholine levels, such as by tetanic stimulation of MHb fibers^17^. Because of the requirement for strong stimulation and the slow kinetics of cholinergic responses, the *volume transmission* mode has been postulated for cholinergic transmission. In this transmission mode, acetylcholine released from cholinergic axons and terminals diffuses in the extracellular space and activates nAChRs on the extrasynaptic cell surface^52,53^. However, in contrast to the cerebral cortex and hippocampus^36,54^, cholinergic terminals in the IPN form specialized synaptic structures called crest-type synapses^55^. At the synapse, two asymmetrical synapses on IPN dendrites are aligned in parallel^56^. A recent 3D reconstruction study using focused ion beam SEM revealed that the nerve endings from the MHb fit into the indentation of U-shaped dendrites of IPN neurons without intervening glial processes^55^. Although the structural significance remains unclear, it can be assumed to increase the efficiency of transmission by increasing the surface area of synaptic contact and the concentration of transmitters such as acetylcholine and glutamate in the synaptic cleft and nearby extrasynaptic space. By post-embedding immunogold, we found here that synaptic junctions of crest synapses are the likely site of glutamatergic signaling, and that α3β4-containing nAChRs are excluded from these junctions. Taken together, the findings suggest that elevated synaptic activities surpassing the threshold level of extracellular acetylcholine concentrations are required to activate widely distributed α3β4-containing nAChRs on MHb axons and extrasynaptic terminals.

While the Ca^2+^ permeability of α3β4-containing nAChRs is low^57^, they exhibit extremely slow desensitization rates and maintain prolonged activation^5^. Therefore, it is conceivable that continued activation of α3β4-containing nAChRs induces depolarization and thereby increases the open probability of voltage-gated Ca^2+^ channels, resulting in an incremental influx of Ca^2+^ and the enhancement of transmitter release^28,58,59^. Hence, α3β4-containing nAChRs could serve as autoreceptors on cholinergic MHb projections to the IPN and operate in a positive feedback mechanism triggered by elevated MHb neuronal activity. Acetylcholine stimulates transmitter release at IPN synapses that lasts for about 2 h, whereas nicotine conversely desensitizes the synapses and decreases transmitter release^60^. That exogenous nicotine and endogenous acetylcholine differ in concentration and duration of action suggests that they may be involved in the complex neuromodulation of the pathways involved in the response to nicotine in vivo.

In both the MHb and IPN, the intensities of α3 immunolabeling were greater in the cytoplasm than in the plasmalemma, indicating the presence of a large cytoplasmic pool of α3β4-containing nAChRs. In a mouse model of chronic nicotine administration, the firing frequency of cholinergic neurons increased upon re-exposure^61^, a phenomenon called nicotine sensitization. This is largely attributed to the functional upregulation of nAChRs in axons and dendrites^28,47^, which has been implicated in nicotine dependence in smokers^29^. Intracellular rather than cell surface nAChRs have been suggested as the pharmacological target of nicotine, which also boosts the cell surface localization of intracellular pools of nAChRs^62,63^. Indeed, recordings from IPN neurons in chronic nicotine-treated mice show increased spontaneous excitatory postsynaptic currents^64^. The abundant cytoplasmic pool of α3β4-containing nAChRs in vMHb neurons may be recruited to the cell surface by nicotine intake. Quantitative expression analysis using chronic nicotine administration models are required to test this concept.

In summary, we produced subunit-specific antibodies and optimized immunohistochemical labeling procedures for localizing endogenous α3β4-containing nAChRs in the MHb–IPN pathway in the adult mouse brain. We detected exclusively high expression of these subunits in MHb neurons as well as broad extrasynaptic expression in the somatodendritic compartment in the MHb and in the axonal compartment in the IPN. These findings demonstrate that these nAChRs are distributed along key pathways involved in nicotine dependence.

## Methods

### Animals

Animal experiments were performed according to the guidelines laid down by the animal experiment committee of Hokkaido University (Protocol #19-0111, #23-0033). Adult (8–12 weeks old) male C57BL/6 mice (Sankyo Lab Service, Tokyo, Japan) were used as the WT mice. Adult (8–12 weeks old) male nAChRα5β4 double-KO mice^33^ (B6.129S7-Chrna5 tm1Mdb Chrnb4 tm1Mdb/Mmmh; RRID: MMRRC_000442-MU, MMRRC, Chapel Hill, USA), which were produced and donated by Arthur Beaudet, M.D., Ph.D., Baylor College of Medicine, were used as negative controls for immunofluorescence and immunoelectron microscopy. Female *Xenopus laevis* frogs were obtained from Sankyo Lab Service and used for oocyte preparation. Three female Japanese white rabbits and Hartley guinea pigs were also obtained from Sankyo Lab Service and used for antibody production.

### Chromogenic in situ hybridization

DIG-labeled riboprobes were prepared to detect target mRNAs^65^. cDNA fragments of mouse nAChRα3 (nucleotide sequence 1090–1680 bp; GenBank Accession Number NM_145129) and nAChRβ4 (1081–1680 bp; NM_148944) were amplified by polymerase chain reaction and subcloned into the pBluescript II plasmid vector. To prepare PFA-fixed brains, mice were deeply anesthetized with pentobarbital (100 mg/kg, i.p.) and perfused transcardially with 60 ml of 4% paraformaldehyde/0.1 M phosphate buffer (PB) (4% PFA, pH 7.2). Brains were then post-fixed in the same fixative at room temperature for 12 h, and Sects. (50-μm-thick) were prepared using a microslicer (VT1000S, Leica Microsystems, Wetzlar, Germany). The following treatments were performed at room temperature using the free-floating method with glass test tubes. Sections were acetylated with 0.25% acetic anhydride/0.1 M triethanolamine hydrochloride solution (pH 8.0) for 10 min, followed by incubation in hybridization solution (50% formamide, 50 mM Tris hydrochloride buffer (pH 7.5), 0.02% Ficoll polyvinylpyrrolidone, 0.02% bovine serum albumin, 0.6 M NaCl, 200 µg/ml tRNA, 1 mM EDTA, 10% dextran sulfate) for 30 min. The hybridization reaction was performed at 63.5 °C for 12 h in the hybridization solution supplemented with cRNA probes at a 1:1000 dilution. Post-hybridization washes were performed sequentially in 5× SSC, 50% formamide/4× SSC, 50% formamide/2× SSC, and 0.1× SSC at 61 °C for 30–40 min each. Sections were then sequentially immersed in NTE buffer (0.5 M sodium chloride, 0.01 M Tris hydrochloric acid buffer (pH 7.5), 5 mM EDTA) for 20 min, in 20 mM iodoacetamide/NTE buffer for 20 min, followed by TNT buffer (0.1 M Tris hydrochloric acid buffer (pH 7.5), 0.15 M NaCl). Next, sections were incubated with DIG blocking solution (1% blocking reagent (Merck, Darmstadt, Germany), 4% normal sheep serum/TNT buffer) for 30 min. Sections were thereafter reacted with alkaline phosphatase-conjugated sheep anti-DIG antibody (Merck)/DIG blocking solution (1:1,000) for 1 h, washed with TNT buffer, and then with 100 mM Tris–HCl (pH 9.5), 100 mM NaCl, 50 mM MgCl_2_. Finally, sections were incubated with NBT/BCIP solution (Merck) overnight at 4 °C to visualize signals. Sections were collected on gelatin-coated glass slides, dehydrated with a graded ethanol series, permeabilized with xylene, and mounted with Entellan (Merck). Sections were photographed under an optical microscope (BZ-X710, Keyence, Osaka) with CFI PlanApo λ(4×/0.2, 10×/0.45, 20×/0.75) objective lenses (Nikon, Tokyo, Japan). For Fig. 1m, measurements were made using MetaMorph software (Molecular Devices, San Jose, CA). Chromogenic images were converted to 8-bit grayscale and their tones were inverted. ROIs were defined manually on specific brain regions and nuclei, and the average signal intensity (in arbitrary units, A.U.) was measured.

### Triple FISH using RNAscope

Under deep anesthesia with isoflurane (5%, Pfizer, Manhattan, NY), brains were rapidly removed, frozen on powdered dry ice, and embedded in O.C.T. compound (Sakura Finetek Japan Co., Ltd., Tokyo, Japan). Coronal frozen sections (20-µm-thick) were prepared using a cryostat (CM1860, Leica Microsystems), mounted on glass slides, air-dried, and processed with the RNAscope Multiplex Fluorescent Reagent kit v2 (Advanced Cell Diagnostics, Newark, CA). The following probes were used: *Chrna3* (α3; Cat. No. 449191-C3), *Chrnb4* (β4; 452,971-C2), *Slc5a7* (CHT1; 439,941 and 439,941-C3), *Slc17a6* (VGluT2; 319,171), *Slc17a7* (VGluT1, 416,631), and *Slc32a1* (VIAAT, 319191-C3) (Advanced Cell Diagnostics). In brief, sections were fixed in 4% PFA, dehydrated through a graded ethanol series, treated with hydrogen peroxide, and then with protease solution at room temperature for 25 min. After washing, the sections were hybridized with probes at 40 °C for 2 h. The sections were then stained with DAPI and mounted onto ProLong Glass (Thermo Fisher Scientific, Waltham, MA). Images were acquired using a confocal laser scanning microscope (FV1200, Evident, Tokyo, Japan) equipped with 405, 473, 559 and 547 nm diode lasers and UPlanXApo (20×/0.80 and 60×/1.42 Oil) objective lenses (Evident). Images were acquired using a range of objective lenses: 20× for Fig. 2a,e,i,m; and 60× for Fig. 2b,f,j,n. Image analysis was performed using MetaMorph software (Molecular Devices). Images were separated into channels and converted to 8-bit grayscale images. The ROI for measurements was established around the DAPI-stained nucleus, manually outlined just beyond the nuclear contour, extending by 20% of the diameter, and the average signal intensity (in the arbitrary unit, A.U.) in each cell was measured. A cut-off threshold (in A.U.) was determined based on background intensities measured in cell-free regions from 6 sections in 2 mice, and set at 5 (A.U.), representing the average plus 4 standard deviations. Positivity for each gene was determined by signals surpassing this threshold. Notably, co-expression of respective genes was inferred when signals for two genes exceeded this criterion.

### Antibodies

Primary antibodies against the following molecules were used: high-affinity choline transporter-1 (CHT1), G protein-coupled receptor 151 (GPR151), microtubule-associated protein 2 (MAP2), nAChR α3 and β4 subunits, postsynaptic density protein-95 (PSD-95), type 1 vesicular glutamate transporter (VGluT1), and vesicular acetylcholine transporter (VAChT). In the present study, we produced α3, β4, and GPR151 antibodies. For expression of glutathione *S*-transferase fusion proteins, we subcloned cDNA fragments encoding mouse α3 subunit, β4 subunit and GPR151 into the pGEX4T-2 plasmid (GE Healthcare, Chicago, IL). Immunization and affinity purification were performed as described previously^43^. In brief, each fusion protein emulsified with Freund's complete adjuvant (Difco, Detroit, MI) was injected subcutaneously into a female Japanese white rabbit and a Hartley guinea pig at intervals of 2–4 weeks. Two weeks after the sixth injection, serum was collected and immunoglobulins specific to antigen peptides were affinity-purified using the antigen-coupled to CNBr-activated Sepharose 4B (Pharmacia Biotech AB). The final antibody concentration is determined using OD_280_ measurement. Information on the molecule, antigen sequence, host species, specificity, reference, NCBI GenBank accession number and RRID are summarized in Table 1. The dilution of antibodies in each experiment was described in individual sections in Methods.

**Table 1:** Details of primary antibodies used.

Molecule	Sequence	#NCBI	Host	Specificity	RRID	References
CHT1	531–580 aa	BC065089.1	Rb, GP, Go	IBb/SL^a^	AB_2571676 2571677 2571678	^68^
GPR151	388–422 aa	NM_181543.2	Rb	SL^b^	AB_3083064	This study
MAP2	927–1104 aa	NM008632.2	Go	SL^c^	AB_2571793	^35^
nAChRα3	345–394 aa	NM_145129.3	GP	Ibx α5β4-KO	AB_3083065	This study
nAChRβ4	206–230 aa	XM_021172517.2	Rb	Ibx α5β4-KO	AB_3083066	This study
panAMPAR	727–745aa	X57497	GP	Ibh	AB_2571610	^69^
PSD-95	1–62 aa	D50621.1	Rb, GP, Go	IBb	AB_2571611 2571612 2920798	^70^
VGluT1	531–560 aa	BC054462.1	Rb, GP, Go	IB/SL^d^	AB_2571616 2,571,617 2,571,618	^71^
VAChT	518–530 aa	AF019145	GP	IB/SL^d^	AB_2736904	^72^

aa, amino acid residues; Go, goat polyclonal antibody; GP, guinea pig polyclonal antibody; IBb, immunoblot with brain homogenates; IBh, immunoblot with HEK293 cells transfected with relevant mouse molecule; IBx, immunoblot with *Xenopus* oocytes transfected with relevant mouse molecule; KO, lack of immunolabeling in knockout brains; Rb, rabbit polyclonal antibody

^a^SL, specific labeling in cholinergic neurons expressing vesicular acetylcholine transporter; ^b^SL, specific labeling in ventral medial habenula and interpeduncular nucleus; ^c^SL, specific labeling in perikaryal and dendrites of various neurons; ^d^SL, specific labeling in glutamatergic axon terminals in the cerebral cortex, hippocampus, striatum, thalamus, and cerebellar cortex. Antibody dilution for each experiment is described in the individual sections of the Methods.

### Immunoblot

The FANTOM clones of mouse *Chnra3* (clone ID: A730007P14) and *Chrnb4* (clone ID 100062851), which were established by the Genome Exploration Research Group, RIKEN GSC, using full-length sequences^66^, were purchased from Dnaform (Yokohama, Japan). Each cDNA was inserted into the pGEM-HE plasmid (Liman et al., 1992), and cRNA was transcribed in vitro using T7 mMessage mMachine (Thermo Fisher Scientific). *Xenopus laevis* were percutaneously anesthetized with tricaine mesylate (Merck), and oocytes were removed from follicular cells using collagenase (Merck) and stored in Ringer’s solution. cRNA (1.0 ng) was injected into each oocyte using a microinjector and glass pipette. Four days after injection, five oocytes were pooled and homogenized in 20 mM Tris/5 mM EDTA pH 8.0 using a Dounce homogenizer. Samples were centrifuged at 16,000 g for 20 s, washed twice, solubilized in TE buffer (20 mM Tris–HCl, 5 mM EDTA, 1% Triton X-100), and agitated at 4 °C for 30 min. Samples were centrifuged at 100,000 g for 20 min, and solubilized proteins were resolved on a 7.5% SDS-PAGE gel, transferred to nitrocellulose membranes, and subjected to immunoblot analysis. After blocking, the membranes were incubated with the primary antibody (1 µg/ml) overnight at room temperature, washed, and then incubated with the secondary antibody (1:10,000; Jackson ImmunoResearch, West Grove, PA) and visualized with ECL Prime (GE Healthcare). Images were acquired with a digital camera (X-A10, Fujifilm, Tokyo, Japan).

### Immunofluorescence

Under deep anesthesia with pentobarbital (100 mg/kg, i.p.), mice were perfused transcardially with 5 ml of saline, followed by 60 ml of glyoxal fixative solution (9% (v/v) glyoxal (Merck), 8% (v/v) acetic acid (pH 4.0))^37,67^. After postfixation overnight, brains were immersed in 30% sucrose solution/0.1 M PB (pH 7.2), and coronal Sects. (50-µm-thick) were prepared using a cryostat (CM1860) and immunostained with the free-floating method using glass test tubes. Phosphate-buffered saline (pH 7.4) containing 0.1% Triton X-100 (PBS-T) was used as incubation and washing buffers. Sections were blocked with 10% normal donkey serum (Jackson ImmunoResearch) for 20 min, then incubated with a mixture of primary antibodies (1 µg/ml each) overnight at room temperature. Thereafter, sections were washed, and incubated with Alexa488, Cy3 and Alexa647-labeled species-specific secondary antibodies (Jackson ImmunoResearch; Thermo Fisher Scientific) for 2 h at room temperature. After washing, sections were mounted on APS-coated glass slides (Matsunami, Osaka, Japan), air-dried, and mounted on ProLong Glass with or without NucBlue stain (Hoechst 33342) (Thermo Fisher Scientific). Photographs were taken with a confocal laser microscope (FV1200, Evident) equipped with 405, 473, 559, and 547 nm diode lasers and UPlanXApo (10×/0.40, 20×/0.80, and 60×/1.42 Oil) and Uplan FL N (40×/1.3 Oil) objective lenses. Images were acquired using a range of objective lenses: 10× for Fig. 3e,f, and 6b; 20× for Fig. 3c,e, 4e, and 6a; 40× for Fig. 4a,f,g,l; and 60× for Fig. 4b,h,i,m,n,o,r, and 6c,d,f. Quantitative analyses were performed using MetaMorph software (Molecular Devices). Images were separated into individual channels and converted to 8-bit grayscale. For Fig. 3g,h, 4c,d, ROIs were defined manually on specific brain regions, nuclei, and compartments, and the average signal intensity (in arbitrary units, A.U.) was measured. For Fig. 4j,k,p,q,s,t, 6e,g,h, immunopositive puncta were semi-automatically detected using the “Inclusive Threshold” and “Create Regions Around Objects” functions. In each ROI, the average signal intensity (in arbitrary units, A.U.) was measured.

### Pre-embedding immunoelectron microscopy

Under deep anesthesia with pentobarbital (100 mg/kg, i.p.), mice were transcardially perfused with 2% PFA/0.1 M PB (pH 7.2) for 10 min, and the brains were removed. Brains were then postfixed for 2 h in the same fixative, and coronal sections (50-µm-thick) were prepared using a vibratome (VT1000S, Leica Microsystems). PBS (pH 7.4) containing 0.1% Tween 20 was used as incubation and washing buffers. Sections were blocked with 10% normal goat serum (Nichirei Bioscience Corporation, Tokyo, Japan) for 20 min, and incubated with guinea pig anti-nAChRα3 antibody (2 µg/ml) for 48 h at room temperature. After washing, sections were incubated with 1.4 nm gold colloid-conjugated anti-guinea pig IgG antibody (1:100; Nanoprobes, Stony Brook, USA) for 24 h at 4 °C. Sections were washed with HEPES Buffer (50 mM HEPES, 200 mM sucrose, 5 N sodium hydroxide) (pH 8.0), and then incubated with silver enhancement reagent (AURION R-Gent SE-EM; AURION, Wageningen, Netherlands) for 1 h. Sections were fixed with 1% osmium tetroxide solution on ice for 15 min, and then stained with 2% uranyl acetate for 15 min, dehydrated in a graded ethanol series and n-butyl glycidyl ether, embedded in epoxy resin, and polymerized at 60 °C for 24 h. Ultrathin sections (~ 80-nm-thick) were prepared with an ultramicrotome (Ultracut UCT, Leica Microsystems) and collected on a copper grid. Finally, sections were treated with 2% uranyl acetate (5 min) and Reynolds’ lead citrate (90 s), and photographed using a transmission electron microscope (H7100, Hitachi, Tokyo, Japan) at magnifications of ×5,000 and ×8,000. Negative films were scanned and converted to digital images for quantitative analysis using MetaMorph (Molecular Devices). Metal particles within 35 nm of the plasmalemma were considered as plasmalemma labeling. The densities of metal particles per unit length of the plasmalemma and per unit area of the cytoplasm were calculated for MHb and IPN cells.

For 3D reconstruction, serial sections (~ 80-nm-thick) were prepared in the plane parallel to the section surface and mounted on indium-tin-oxide-coated glass slides (IT5-111-50, NANOCS, Boston, MA). Sections were successively stained with 2% uranyl acetate and lead citrate. After washing, colloidal graphite (Ted Pella Inc, Redding, CA) was pasted on the glass slides to surround the ribbons. Images were acquired using a SEM with a backscattered electron beam detector at an accelerating voltage of 1.0 kV and a magnification of ×9000 (SU8240, Hitachi High Technologies, Tokyo, Japan). SEM images were loaded into Image Pro 10 (Media Cybernetics, Rockville, MD), aligned, and the structures of interest were segmented by delineating their boundary contours to create 3D surface renderings.

### Post-embedding immunoelectron microscopy

Under deep anesthesia with pentobarbital (100 mg/kg, i.p.), mice were transcardially perfused with 4% PFA/0.1 M PB (pH 7.2) containing 0.1% glutaraldehyde for 10 min. The brains were then removed and postfixed for 2 h in the same fixative, and coronal Sects. (300-µm-thick) were prepared using a vibratome (VT1000S, Leica Microsystems). The sections were cryoprotected with 30% glycerol in PB and frozen rapidly with liquid propane in the EM CPC unit (Leica Microsystems). Frozen sections were transferred to the AFS freeze-substitution unit (Leica Microsystems), where freeze substitution proceeded as follows: 0.5% uranyl acetate in methanol at − 90 °C for 24 h; the temperature was increased at 4 °C/h to − 45 °C; 100% methanol at − 45 °C for 30 min (3 times). The following steps, including the infiltration with LR gold (Electron Microscopy Sciences, Hatfield, PA), were conducted at − 25 °C. Samples were immersed successively with a mixture of methanol and LR Gold (1:1) for 1 h; 1:2 for 1 h; pure LR Gold for 1 h; and a mixture of pure LR Gold supplemented with 0.1% BENZIL (Electron Microscopy Sciences) overnight. After transferring to fresh LR Gold with 0.1% BENZIL, samples were polymerized under UV light for 48 h. Ultrathin sections (90-nm-thick) were mounted on nickel grids, etched with saturated sodium ethoxide solution for 1–5 s, and blocked with 2% normal goat serum (Nichirei) in 0.03% Triton X-100 in Tris-buffered saline (TTBS, pH 7.4) for 20 min. The sections on the grids were then incubated with primary antibodies (20 µg/ml) diluted in 1% normal goat serum in TTBS overnight, washed with TTBS, and thereafter incubated with colloidal gold-conjugated (10 nm) anti-rabbit or anti-guinea pig IgG (1:100, British BioCell International) for 2 h. After extensive washing in TTBS, grids were rinsed with distilled water and stained with 1% OsO_4_ for 15 min, followed by 2% uranyl acetate for 5 min and Reynolds’ lead citrate for 1 min. Sections were photographed using a transmission electron microscope (JEM1400, JEOL, Tokyo, Japan) at a magnification of ×20,000.

### Statistical analysis

All values are expressed as the mean ± SEM (where n = number of analyzed neuronal profiles, cells, or ROIs, unless otherwise noted). Graphing and statistical tests were performed using GraphPad Prism 10. Normality was determined with D’Agostino & Pearson, Anderson–Darling, Shapiro–Wilk, or one sample Kolmogorov–Smirnov tests. In all cases, the assumption of normality is not met for all groups, statistics were performed using non-parametric tests. Comparisons of two groups were performed using a two-tailed unpaired Mann–Whitney U-test, the nonparametric equivalent of the t-test. For three or more group comparisons, we conducted the Kruskal–Wallis test. If significant differences were detected, Dunn’s test assessed post-hoc multiple comparisons. In all figures, statistical significance is presented as * *p* < 0.05, ** *p* < 0.01, *** *p* < 0.001.

### Data availability

The datasets for this study are available from the corresponding author upon reasonable request. Antibodies used in this study that are presented with RRID, can be purchased from Nittobo Medical Co., Ltd. (https://nittobo-nmd.co.jp/english/product/product_list.php). Other antibodies are also available from the corresponding author upon reasonable request.

### Supplementary Information

Supplementary Information.

## References

[1] Albuquerque EX, Pereira EF, Alkondon M, Rogers SW (2009). Mammalian nicotinic acetylcholine receptors: From structure to function. Physiol. Rev..

[2] Bouzat C, Sine SM (2018). Nicotinic acetylcholine receptors at the single-channel level. Br. J. Pharmacol..

[3] Sargent PB (1993). The diversity of neuronal nicotinic acetylcholine receptors. Annu. Rev. Neurosci..

[4] Zoli M, Pistillo F, Gotti C (2015). Diversity of native nicotinic receptor subtypes in mammalian brain. Neuropharmacology.

[5] Fenster CP, Rains MF, Noerager B, Quick MW, Lester RA (1997). Influence of subunit composition on desensitization of neuronal acetylcholine receptors at low concentrations of nicotine. J. Neurosci..

[6] Rose JE (2007). Multiple brain pathways and receptors underlying tobacco addiction. Biochem. Pharmacol..

[7] Amos CI (2008). Genome-wide association scan of tag SNPs identifies a susceptibility locus for lung cancer at 15q25.1. Nat. Genet..

[8] Jensen KP (2015). A CHRNA5 smoking risk variant decreases the aversive effects of nicotine in humans. Neuropsychopharmacology.

[9] Thorgeirsson TE (2008). A variant associated with nicotine dependence, lung cancer and peripheral arterial disease. Nature.

[10] Frahm S (2011). Aversion to nicotine is regulated by the balanced activity of β4 and α5 nicotinic receptor subunits in the medial habenula. Neuron.

[11] Elayouby KS (2021). α3* nicotinic acetylcholine receptors in the habenula-interpeduncular nucleus circuit regulate nicotine intake. J. Neurosci..

[12] Fowler CD, Kenny PJ (2014). Nicotine aversion: Neurobiological mechanisms and relevance to tobacco dependence vulnerability. Neuropharmacology.

[13] Hikosaka O (2010). The habenula: From stress evasion to value-based decision-making. Nat. Rev. Neurosci..

[14] Yamaguchi T, Danjo T, Pastan I, Hikida T, Nakanishi S (2013). Distinct roles of segregated transmission of the septo-habenular pathway in anxiety and fear. Neuron.

[15] Aizawa H, Kobayashi M, Tanaka S, Fukai T, Okamoto H (2012). Molecular characterization of the subnuclei in rat habenula. J. Comp. Neurol..

[16] Wagner F, Stroh T, Veh RW (2014). Correlating habenular subnuclei in rat and mouse by using topographic, morphological, and cytochemical criteria. J. Comp. Neurol..

[17] Ren J (2011). Habenula “cholinergic” neurons co-release glutamate and acetylcholine and activate postsynaptic neurons via distinct transmission modes. Neuron.

[18] Contestabile A (1987). Topography of cholinergic and substance P pathways in the habenulo-interpeduncular system of the rat. An immunocytochemical and microchemical approach. Neuroscience.

[19] Herkenham M, Nauta WJ (1979). Efferent connections of the habenular nuclei in the rat. J. Comp. Neurol..

[20] Lima LB (2017). Afferent and efferent connections of the interpeduncular nucleus with special reference to circuits involving the habenula and raphe nuclei. J. Comp. Neurol..

[21] Ogawa SK, Cohen JY, Hwang D, Uchida N, Watabe-Uchida M (2014). Organization of monosynaptic inputs to the serotonin and dopamine neuromodulatory systems. Cell Rep..

[22] Groenewegen HJ, Ahlenius S, Haber SN, Kowall NW, Nauta WJ (1986). Cytoarchitecture, fiber connections, and some histochemical aspects of the interpeduncular nucleus in the rat. J. Comp. Neurol..

[23] Sutherland RJ (1982). The dorsal diencephalic conduction system: a review of the anatomy and functions of the habenular complex. Neurosci. Biobehav. Rev..

[24] Quick MW, Ceballos RM, Kasten M, McIntosh JM, Lester RA (1999). α3β4 subunit-containing nicotinic receptors dominate function in rat medial habenula neurons. Neuropharmacology.

[25] Salas R, Pieri F, De Biasi M (2004). Decreased signs of nicotine withdrawal in mice null for the β4 nicotinic acetylcholine receptor subunit. J. Neurosci..

[26] Wada E (1989). Distribution of α2, α3, α4, and β2 neuronal nicotinic receptor subunit mRNAs in the central nervous system: a hybridization histochemical study in the rat. J. Comp. Neurol..

[27] Winzer-Serhan UH, Leslie FM (1997). Codistribution of nicotinic acetylcholine receptor subunit α3 and β4 mRNAs during rat brain development. J. Comp. Neurol..

[28] Arvin MC (2019). Chronic nicotine exposure alters the neurophysiology of habenulo-interpeduncular circuitry. J. Neurosci..

[29] Shih PY (2014). Differential expression and function of nicotinic acetylcholine receptors in subdivisions of medial habenula. J. Neurosci..

[30] Dineley-Miller K, Patrick J (1992). Gene transcripts for the nicotinic acetylcholine receptor subunit, β4, are distributed in multiple areas of the rat central nervous system. Brain Res. Mol. Brain Res..

[31] Mu R (2022). A cholinergic medial septum input to medial habenula mediates generalization formation and extinction of visual aversion. Cell Rep..

[32] Xu W (1999). Megacystis, mydriasis, and ion channel defect in mice lacking the α3 neuronal nicotinic acetylcholine receptor. Proc. Natl. Acad. Sci. U. S. A..

[33] Kedmi M, Beaudet AL, Orr-Urtreger A (2004). Mice lacking neuronal nicotinic acetylcholine receptor β4-subunit and mice lacking both α5- and β4-subunits are highly resistant to nicotine-induced seizures. Physiol. Genomics.

[34] Andres KH, von Düring M, Veh RW (1999). Subnuclear organization of the rat habenular complexes. J. Comp. Neurol..

[35] Miura E (2006). Expression and distribution of JNK/SAPK-associated scaffold protein JSAP1 in developing and adult mouse brain. J. Neurochem..

[36] Yamasaki M, Matsui M, Watanabe M (2010). Preferential localization of muscarinic M1 receptor on dendritic shaft and spine of cortical pyramidal cells and its anatomical evidence for volume transmission. J. Neurosci..

[37] Konno K, Yamasaki M, Miyazaki T, Watanabe M (2023). Glyoxal fixation: An approach to solve immunohistochemical problem in neuroscience research. Sci. Adv..

[38] Berthold M, Collin M, Sejlitz T, Meister B, Lind P (2003). Cloning of a novel orphan G protein-coupled receptor (GPCR-2037): In situ hybridization reveals high mRNA expression in rat brain restricted to neurons of the habenular complex. Brain Res. Mol. Brain Res..

[39] Broms J, Antolin-Fontes B, Tingstrom A, Ibanez-Tallon I (2015). Conserved expression of the GPR151 receptor in habenular axonal projections of vertebrates. J. Comp. Neurol..

[40] Jones IW, Wonnacott S (2005). Why doesn't nicotinic ACh receptor immunoreactivity knock out?. Trends Neurosci..

[41] Moser N (2007). Evaluating the suitability of nicotinic acetylcholine receptor antibodies for standard immunodetection procedures. J. Neurochem..

[42] Gahring LC, Persiyanov K, Rogers SW (2004). Neuronal and astrocyte expression of nicotinic receptor subunit beta4 in the adult mouse brain. J. Comp. Neurol..

[43] Watanabe M, Lujan R, Ciruela F (2021). Production of high-quality antibodies for the study of receptors and ion channels. Receptor and Ion Channel Detection in the Brain.

[44] McDonough J, Francis N, Miller T, Deneris ES (2000). Regulation of transcription in the neuronal nicotinic receptor subunit gene cluster by a neuron-selective enhancer and ETS domain factors. J. Biol. Chem..

[45] Matta JA (2017). NACHO mediates nicotinic acetylcholine receptor function throughout the brain. Cell Rep..

[46] Contestabile A, Fonnum F (1983). Cholinergic and GABAergic forebrain projections to the habenula and nucleus interpeduncularis: surgical and kainic acid lesions. Brain Res..

[47] Banala S (2018). Photoactivatable drugs for nicotinic optopharmacology. Nat Methods.

[48] Schäfer MK, Eiden LE, Weihe E (1998). Cholinergic neurons and terminal fields revealed by immunohistochemistry for the vesicular acetylcholine transporter. I. Central nervous system. Neuroscience.

[49] Woolf NJ (1991). Cholinergic systems in mammalian brain and spinal cord. Prog. Neurobiol..

[50] Herkenham M, Nauta WJ (1977). Afferent connections of the habenular nuclei in the rat. A horseradish peroxidase study, with a note on the fiber-of-passage problem. J. Comp. Neurol..

[51] Brown DA, Docherty RJ, Halliwell JV (1983). Chemical transmission in the rat interpeduncular nucleus in vitro. J. Physiol..

[52] Sarter M, Parikh V, Howe WM (2009). Phasic acetylcholine release and the volume transmission hypothesis: Time to move on. Nat. Rev. Neurosci..

[53] Dani JA, Bertrand D (2007). Nicotinic acetylcholine receptors and nicotinic cholinergic mechanisms of the central nervous system. Annu. Rev. Pharmacol. Toxicol..

[54] Descarries L, Mechawar N (2000). Ultrastructural evidence for diffuse transmission by monoamine and acetylcholine neurons of the central nervous system. Prog. Brain Res..

[55] Parajuli LK, Wako K, Maruo S, Kakuta S, Koike M (2020). Unique synaptic topography of crest-type synapses in the interpeduncular nucleus. Biochem. Biophys. Res. Commun..

[56] Lenn NJ (1976). Synapses in the interpeduncular nucleus: Electron microscopy of normal and habenula lesioned rats. J. Comp. Neurol..

[57] Fucile S (2004). Ca^2+^ permeability of nicotinic acetylcholine receptors. Cell Calcium.

[58] Girod R, Barazangi N, McGehee D, Role LW (2000). Facilitation of glutamatergic neurotransmission by presynaptic nicotinic acetylcholine receptors. Neuropharmacology.

[59] Grady SR (2009). Rodent habenulo-interpeduncular pathway expresses a large variety of uncommon nAChR subtypes, but only the α3β4* and α3β3β4* subtypes mediate acetylcholine release. J. Neurosci..

[60] Girod R, Role LW (2001). Long-lasting enhancement of glutamatergic synaptic transmission by acetylcholine contrasts with response adaptation after exposure to low-level nicotine. J. Neurosci..

[61] Görlich A (2013). Reexposure to nicotine during withdrawal increases the pacemaking activity of cholinergic habenular neurons. Proc. Natl. Acad. Sci. U. S. A..

[62] Corringer PJ, Sallette J, Changeux JP (2006). Nicotine enhances intracellular nicotinic receptor maturation: A novel mechanism of neural plasticity?. J. Physiol. Paris.

[63] Lester HA (2009). Nicotine is a selective pharmacological chaperone of acetylcholine receptor number and stoichiometry Implications for drug discovery. AAPS J..

[64] Zhao-Shea R, Liu L, Pang X, Gardner PD, Tapper AR (2013). Activation of GABAergic neurons in the interpeduncular nucleus triggers physical nicotine withdrawal symptoms. Curr. Biol..

[65] Yamasaki M (2001). 3-Phosphoglycerate dehydrogenase, a key enzyme for l-serine biosynthesis, is preferentially expressed in the radial glia/astrocyte lineage and olfactory ensheathing glia in the mouse brain. J. Neurosci..

[66] Carninci P (2005). The transcriptional landscape of the mammalian genome. Science.

[67] Richter KN (2018). Glyoxal as an alternative fixative to formaldehyde in immunostaining and super-resolution microscopy. EMBO J..

[68] Narushima M (2007). Tonic enhancement of endocannabinoid-mediated retrograde suppression of inhibition by cholinergic interneuron activity in the striatum. J. Neurosci..

[69] Fukaya M (2006). Abundant distribution of TARP gamma-8 in synaptic and extrasynaptic surface of hippocampal neurons and its major role in AMPA receptor expression on spines and dendrites. Eur. J. Neurosci..

[70] Fukaya M, Watanabe M (2000). Improved immunohistochemical detection of postsynaptically located PSD-95/SAP90 protein family by protease section pretreatment: A study in the adult mouse brain. J. Comp. Neurol..

[71] Miyazaki T, Fukaya M, Shimizu H, Watanabe M (2003). Subtype switching of vesicular glutamate transporters at parallel fibre-Purkinje cell synapses in developing mouse cerebellum. Eur. J. Neurosci..

[72] Nakamura M (2004). Signaling complex formation of phospholipase Cbeta4 with metabotropic glutamate receptor type 1alpha and 1,4,5-trisphosphate receptor at the perisynapse and endoplasmic reticulum in the mouse brain. Eur. J. Neurosci..

