# Supplementary material for: Abundant extrasynaptic expression of α3β4-containing nicotinic acetylcholine receptors in the medial habenula–interpeduncular nucleus pathway in mice
Source: Sci Rep. 2024 Jun 20;14:14193. doi: 10.1038/s41598-024-65076-3 (PMC11189931; doi:10.1038/s41598-024-65076-3)
Supplement: Supplementary file 1 — Supplementary Information. [file 41598_2024_65076_MOESM1_ESM.pdf]

Supplemental information

Tsuzuki and Yamasaki et al.

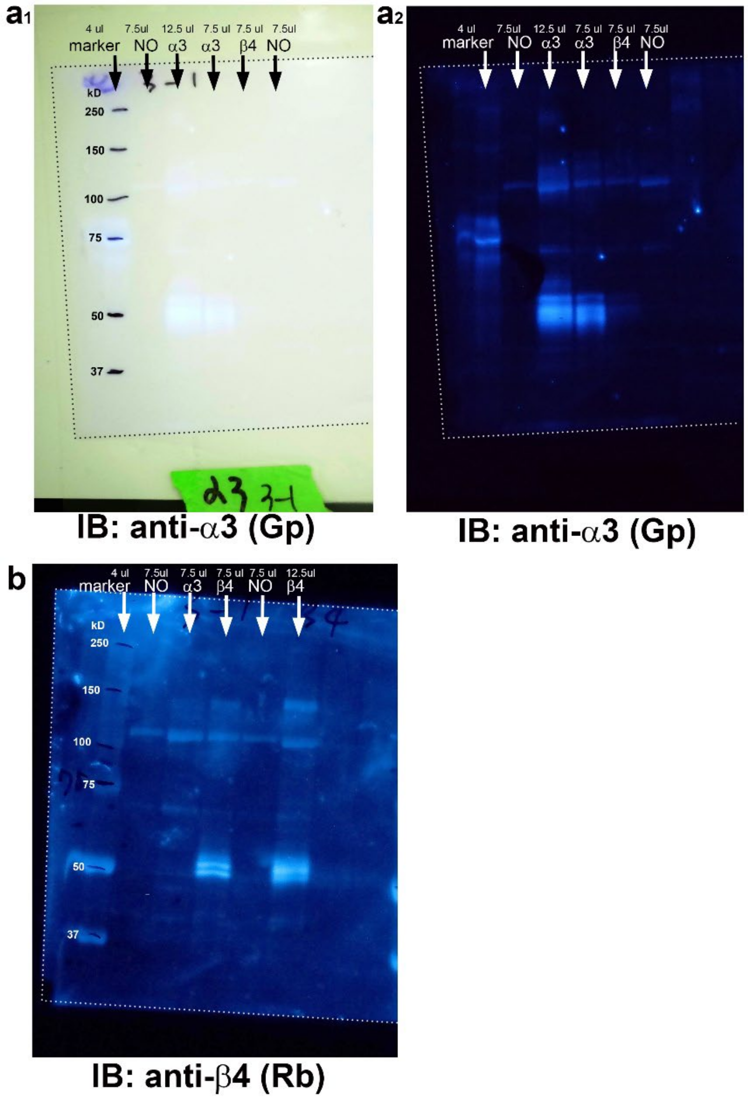

Supplemental Figure S1. Original immunoblot membrane, related to Fig. 3a, b.

(**a**) A bright-field image (**a**$_1$) and a dark-field image (**b**$_1$) of a chemiluminescence immunoblot with the guinea pig anti-α3 antibody. (**b**) A dark-field image of chemiluminescence immunoblot with the rabbit anti-β4 antibody. The immunoblot membranes were cut prior to incubation with the indicated primary antibodies. A protein marker (Precision Plus Protein, All Blue Pre-Stained Protein Standards #1610373, Bio-Rad) and oocyte lysate with no injected cRNA (NO), α3, β4 was loaded. Loading volume is indicated at the top. The images were converted to grayscale, inverted tones, cropped and used for Fig. 3a and b.

Supplementary Table S1. Number of observations and statistical testing, related to Fig. 4

The sample size that has been used for statistical testing is shown in bold for each data set.

**Panel**	**Sample size**	**Statistical tests and p-values, not provided in the figure, figure legend, or results section**
(c, d)	n = **20–32 ROIs**/3 images/3 mice.	shown in the figure and figure legend
(j)	n = **6–9 images**/3 mice.	Kruskal-Wallis test and Dunn's multiple comparison test.<br>dMHb vs. vMHb, $p = 0.99$<br>dMHb vs. mLHb, $p = 0.59$<br>dMHb vs. lLHb, $p = 0.10$<br>mLHb vs. lLHb, $p = 0.99$
(k)	n = **78–562 ROIs**/6 images/3 mice.	Kruskal-Wallis test and Dunn's multiple comparison test.<br>mLHb vs. lLHb, $p = 0.15$
(p)	n = **22–40 ROIs**/3 images/3 mice	Kruskal-Wallis test and Dunn's multiple comparison test.<br>PSD-95 vs. VGluT1, $p = 0.99$<br>PSD-95 vs. Rt.α3, $p = 0.10$<br>VGluT1 vs. Rt.α3, $p = 0.67$
(q)	n = **20–38 ROIs**/3 images/3 mice.	Kruskal-Wallis test and Dunn's multiple comparison test.<br>PSD-95 vs. VGluT1, $p = 0.99$<br>PSD-95 vs. Rt.β4, $p = 0.10$<br>VGluT1 vs. Rt.β4, $p = 0.60$
(s)	n = **19–23 ROIs**/3 images/3 mice;	Kruskal-Wallis test and Dunn's multiple comparison test.<br>α3 vs. β4, $p = 0.99$<br>PSD-95 vs. Rt.α3, $p = 0.99$
(t)	n = **16–23 ROIs**/3 images/3 mice.	Kruskal-Wallis test and Dunn's multiple comparison test.<br>α3 vs. β4, $p = 0.99$<br>PSD-95 vs. Rt.β4, $p = 0.43$

Supplementary Table S2. Number of observations and statistical testing, related to Fig. 6

The sample size that has been used for statistical testing is shown in bold for each data set.

**Panel**	**Sample size**	**Statistical tests and p-values, not provided in the figure, figure legend, or results section**
(e)	n = **17–34 ROIs**/3 images/3 mice	shown in the figure and results section
(g)	n = **72–297 ROIs**/3 images/3 mice	Kruskal-Wallis test and Dunn's multiple comparison test.<br>α3 vs. β4, $p = 0.05$
(h)	n = **142–351 ROIs**/3 images/3 mice	Kruskal-Wallis test and Dunn's multiple comparison test.<br>α3 vs. β4, $p = 0.06$
